# Error State Kalman Filter for Integrated Attitude Estimation Based on Data Fusion of MIMU Inertial Array and Magnetometer

**DOI:** 10.3390/mi17091064

**Published:** 2026-09-08

**Authors:** Liang Xue, Jixiang Lu, Guangbin Cai, Bo Yang, Xinguo Wang

**Affiliations:** Department of Control Engineering, Rocket Force University of Engineering, Hongqing Town, No. 2 Tongxin Road, Xi’an 710025, China; lujixiang7@163.com (J.L.); cgb0712@163.com (G.C.); yangbo8093@sina.com (B.Y.); wxgsea@163.com (X.W.)

**Keywords:** MEMS sensor, inertial measurement array, magnetometer, attitude estimation, error state Kalman filter

## Abstract

Attitude estimation has increasingly relied on MEMS inertial measurement units (IMUs) owing to the low cost and miniature size, but the inherent high random noise and error accumulation limit long-term measurement accuracy. This article proposes an integrated attitude estimation algorithm based on data fusion from a MIMU inertial array and magnetometer to address this challenge. First, a redundant inertial array is constructed using homogeneous gyroscopes, and a Kalman filter (KF) is designed to fuse output signals from multiple gyroscopes to estimate true angular rate. Second, an integrated error state Kalman filter (ESKF) for the MIMU/magnetometer system is developed. Using the attitude quaternion calculated by the strapdown inertial solution as the nominal state and combining it with measurements from the accelerometer and magnetometer as observations, the attitude error is estimated and corrected. Both simulations and field experiments were conducted to validate the effectiveness of the proposed algorithm. The experimental results show that the ESKF algorithm performs best in estimation accuracy and addresses the issue of error accumulation and fluctuation. In particular, the Root Mean Square Error (RMSE) of the ESKF algorithm was significantly reduced, with the roll angle reduced by 55.34%, the pitch angle by 30.25%, and the yaw angle by 55.71%.

## 1. Introduction

Accurate and high-speed attitude estimation has emerged as a paramount prerequisite, driven by the booming proliferation of autonomous systems like robots, unmanned aerial vehicles (UAVs), and unmanned ground vehicles (UGVs) [1]. Recent years have witnessed substantial enhancements in the precision and dependability of low-cost micro inertial measurement units (MIMUs), driven by the continuous evolution of MEMS fabrication processes [2]. Due to cost and resource constraints, high-precision inertial navigation systems are not suitable for such scenarios. For attitude measurement in unmanned systems, the low-cost MEMS IMU remains the most suitable option. A MEMS IMU consists of a three-axis gyroscope and a three-axis accelerometer, providing comprehensive navigation data. A vibrating MEMS gyroscope detects the Coriolis force induced by angular motion, which is measured as a capacitance change proportional to the angular rate. A accelerometer measures acceleration via the capacitance change induced by the displacement of its proof mass under external force. The MEMS IMU offers a high degree of autonomy and stealth and is resistant to external disturbances [3]. The gyroscope obtains real-time attitude angle information by measuring and integrating the angular rate of the platform, while the accelerometer can also determine the platform’s attitude with the assistance of the gravity vector. Nevertheless, inherent constraints in MEMS fabrication processes result in substantially elevated random noise levels in MEMS gyroscopes compared to fiber-optic and laser counterparts. Consequently, the rapid accumulation of errors during attitude estimation renders them inadequate for prolonged operational tasks [4]. MEMS accelerometers do not suffer from cumulative integration errors during long-term attitude estimation, but their measured values represent the specific force acting on the platform, making them susceptible to dynamic acceleration, which can cause attitude estimation errors to diverge.

To address the above problems, two approaches are commonly used: improving the accuracy of the MEMS IMU and multi-sensor data fusion [5]. The overall measurement accuracy of an IMU is predominantly dictated by the angular rate accuracy of its MEMS gyroscope. MEMS gyroscopes typically contain rotating or vibrating components that measure angular rate using the Coriolis effect, making them more susceptible to noise and disturbance. The implementation of gyro array technology represents a potent strategy for enhancing the measurement accuracy of MEMS gyroscopes. Compared to other high-precision devices, it is low-cost and lightweight, making it suitable for resource-constrained unmanned platforms. Gyro array technology was first proposed by Bayard et al. in 2003 [6]. The underlying principle of this method relies on utilizing KF to combine the outputs of redundant MEMS gyroscopes, thereby generating a virtual gyroscope with optimized performance. Literature [7] used a Kalman filter to fuse multiple uncorrelated MEMS gyroscopes. By adjusting the parameter $q_{\omega }$, the accuracy was improved beyond that of simple averaging. The method was validated using six gyroscopes in experiments, resulting in a significant reduction in noise and bias instability. Literature [8] proposes a statistical method for analyzing the actual correlation coefficients of a MEMS gyro array to address the problem of determining the noise covariance matrix. Experiments with a four-gyro array show that negative noise correlation can improve accuracy. In [9], a two-stage decoupling fusion strategy for unknown disturbances was proposed for the first time. Through local estimation and global weighted fusion, bias accuracy is maintained under disturbances. In [10], the measurement noise covariance of the virtual gyroscope is optimized by employing an improved Sage-Husa adaptive Kalman filter. Literature [11] introduces a novel fusion strategy that surpasses the traditional accuracy limit associated with constant weight fusion. By dynamically adjusting weights, this approach effectively reduces standard deviation without compromising bandwidth. The gyroscope array has been extensively studied as an effective approach to resolving the trade-off between accuracy and cost. Other related techniques involve long short-term memory (LSTM) networks [12,13], extreme learning machines [14], attention mechanisms [15], and reinforcement learning [16]. Currently, there has been little research on gyroscope arrays used directly for navigation. Although the gyroscope array can reduce random noises through KF, its residual noise cannot be eliminated, and the resulting error accumulation must therefore be corrected using external references.

Multi-sensor data fusion is another key method for addressing the accumulation of attitude errors and improving system accuracy and robustness. Currently, there are various combinations of inertial sensors with GNSS, magnetic sensors and other types of sensors. Given the differences in characteristics of MEMS IMUs and magnetic sensors, integrating inertial information and magnetic field measurements using fusion algorithms typically yields better attitude estimation accuracy and stability. The most commonly used sensors are accelerometers and magnetometers, which provide a relatively stable long-term reference for correcting the integration error in attitude estimation. Literature [17] proposes a segmented angular rate estimation method using the IMU array. An extended Kalman filter is used for low dynamic conditions, while the Levenberg–Marquardt iterative method is used for high-dynamic conditions. To mitigate magnetic noise coupling, Literature [18] presented a novel decoupled extended KF, which utilizes dual independent quaternions to estimate attitude and heading, respectively. Literature [19] detects magnetic field trend changes using a sliding window to determine magnetometer data fusion, significantly improving attitude accuracy and stability under magnetic disturbances. Literature [20] combines angular accelerometers with low-cost gyroscopes and uses deep learning to automatically learn optimal noise covariance parameters from raw data. Literature [21] uses a nine-axis IMU to estimate attitude and proposes a magnetic disturbance detection and adaptive gradient descent algorithm based on quaternion similarity. These methods all leverage the complementary strengths of heterogeneous sensors to improve the accuracy of attitude estimation. A nine-axis MEMS IMU typically includes a gyroscope, accelerometer and magnetometer, which can provide comprehensive navigation data, including angular rate, acceleration, and geomagnetic field measurements. By designing an appropriate fusion algorithm, the accuracy of attitude estimation can be significantly improved. An extensive literature review reveals that most existing studies address isolated aspects, such as optimizing only the array fusion strategy or improving only the navigation algorithm, while few integrate the full pipeline and validate it through real-world experiments.

To address the challenges of cost and resource-constrained unmanned systems, this paper introduces a data fusion algorithm that combines a MIMU inertial array with a magnetometer for robust attitude estimation. First, a Kalman filter is applied to fuse the outputs of the redundant sensor array, which is built using homogeneous gyroscopes, in order to estimate the true angular rate. In the second stage, a fusion algorithm based on an error state Kalman filter is established for the MIMU/magnetometer system, aiming to determine the quaternion error corresponding to the strap-down inertial navigation solution. Finally, attitude estimation with higher accuracy is achieved, and the performance of the integrated ESKF algorithm regarding signal fusion and attitude measurement is validated through comprehensive simulations and experiments.

## 2. Data Fusion Algorithm for Inertial Array

### 2.1. Sensor Error Models for Integrated System

The measurements from MEMS gyroscopes contain various types of random error, such as Angular Random Walk (ARW) and Rate Random Walk (RRW). For measurement error models, while incorporating more error terms can certainly improve accuracy, it also increases model complexity, which is not conducive to the subsequent design of filtering algorithms. The predominant sources of stochastic error, as identified through the evaluation of actual measurement data, are angle random walk and rate random walk. Therefore, the error model for MEMS gyroscopes considering ARW and RRW can be written as:

(1) ωimb=ωr+biω+niωb˙iω=wbi

where ωimb is the output signal of *i*th gyroscope in body coordinate system, $\omega^{r}$ is the true angular rate, $b_{i}^{\omega }$ is rate random walk, $n_{i}^{\omega }$ is Gaussian white noise representing angle random walk, and $w_{b_{i}}$ is the driving Gaussian white noise for rate random walk.

To directly estimate the true angular rate, ω must be modeled. To capture the dynamic evolution of the true angular rate, a first-order Markov formulation is utilized:

(2) $${\dot{\omega }}^{r}=-\frac{1}{\tau }\cdot \omega^{r}+n_{\omega },$$

where τ is the correlation time constant, $n_{\omega }$ contains Gaussian white noises with variance $q_{\omega }$, and the magnitude of $q_{\omega }$ characterizes the dynamic variation of input angular rate.

In addition to the redundant gyro array used to measure angular rate, the system also includes an accelerometer and magnetometer. The accelerometer is used to measure the linear acceleration of the carrier, and the magnetometer is used to measure the magnetic field strength of the surrounding environment. This forms the MIMU/magnetometer system that provides comprehensive data for attitude calculation. The error models for the accelerometer and magnetometer are presented below.

(3) amb=ar+na+g,

(4) mmb=mr+bm+d,

where amb and mmb represent the measurements from the accelerometer and magnetometer, $a^{r}$ and $m^{r}$ are the true reference of the accelerometer and magnetometer, $n^{a}$ is the Gaussian white noise, $b^{m}$ is the bias error of the magnetometer, g is the gravity acceleration, d is the magnetic disturbance vector. The accelerometer and magnetometer output measurement signals after calibration, which are normalized for subsequent attitude estimation.

### 2.2. Filter Algorithm Design for MIMU Array Fusion

Fusing the three-axis angular rate output signals from the inertial array is key to the first-stage filter algorithm, which is used to suppress random noise in the measurement signals and reduce error accumulation during attitude estimation. Multiple gyroscopes are installed in a parallel configuration, and installation errors have been compensated for through calibration. Using the Kalman filter architecture, for an inertial array containing *N* gyroscopes, the true angular rate $\omega^{r}$ relative to the carrier frame and the gyroscope zero bias are selected to form the state vector:

(5) $$X_{1}={\left[{\left(\omega^{r}\right)}^{T},b_{1}^{T},b_{2}^{T},\ldots ,b_{N}^{T}\right]}^{T}\in R^{3+3N},$$

where $\omega^{r}={\left[\omega_{x}^{r},\omega_{y}^{r},\omega_{z}^{r}\right]}^{T}$ is true angular rate vector, $b_{i}={\left[b_{ix},b_{iy},b_{iz}\right]}^{T}$ is the bias vector of *i*th gyro. Noise vector $n_{\omega }=\left[n_{\omega x},n_{\omega y},n_{\omega z}\right]$ denotes as the driving noise of $\omega^{b}$.

Based on the error model established, by treating the gyro array output as the measurement for KF, the state-space model can be obtained as follows:

(6) $$\left\{\begin{array}{c} {\dot{X}}_{1}\left(t\right)=F_{1}\cdot X_{1}\left(t\right)+W_{1}\left(t\right)\\ Z_{1}\left(t\right)=H_{1}\cdot X_{1}\left(t\right)+V_{1}\left(t\right) \end{array}\right.,$$

where $F_{1}$ and $H_{1}$ are coefficient matrices, the measurement vector $Z_{1}\left(t\right)$ consists of the three-axis output signals from the gyro array, process noise $W_{1}={\left[n_{\omega }^{T},w_{b1}^{T},\ldots ,w_{bN}^{T}\right]}^{T}$, $w_{bi}^{T}=\left[w_{bix},w_{biy},w_{biz}\right]$, measurement noise $V_{1}={\left[v_{1}^{T},\ldots ,v_{N}^{T}\right]}^{T}$, $v_{1}^{T}={\left[n_{ix},n_{iy},n_{iz}\right]}^{T}$, all noise vectors are Gaussian white noise and satisfy the following relationship:

(7) $$\left\{\begin{array}{c} E\left[W_{1}\left(t\right)W_{1}^{T}\left(t+\tau \right)\right]=Q_{1}\delta \left(\tau \right)\\ E\left[V_{1}\left(t\right)V_{1}^{T}\left(t+\tau \right)\right]=R_{1}\delta \left(\tau \right) \end{array}\right.,$$

where $Q_{1}$ and $R_{1}$ are noise covariance matrices.

The coefficient matrices $F_{1}$ and $H_{1}$ are shown as follows:

(8) $$F_{1}=\left[\begin{array}{cc} -\frac{1}{\tau }\cdot I_{3} & 0_{3\times 3N}\\ 0_{3N\times 3} & 0_{3N\times 3N} \end{array}\right],$$

(9) $$H_{1}=\left[1_{N}⊗I_{3},I_{3N}\right],$$

where $1_{N}$ is *N*-dimensional all-ones column vector, I represents identity matrix with appropriate dimension, ⊗ in Equation (9) is the Kronecker product. In following sections, ⊗ denotes quaternion multiplication. After discretizing the state-space model (6), the estimate of the true angular rate can be obtained using a discrete Kalman filter:

(10) $$P_{k|k-1}=F_{k,k-1}P_{k-1}F_{k,k-1}^{T}+Q_{k-1},$$

(11) $$K_{k}=P_{k|k-1}H_{k}^{T}{\left(H_{k}P_{k|k-1}H_{k}^{T}+R_{k}\right)}^{-1},$$

(12) $$P_{k}=\left(I-K_{k}H_{k}\right)P_{k|k-1}{\left(I-K_{k}H_{k}\right)}^{T}+K_{k}R_{k}K_{k}^{T},$$

(13) $${\hat{X}}_{k}=F_{k,k-1}{\hat{X}}_{k-1}+K_{k}\left(Z_{k}-H_{k}F_{k,k-1}{\hat{X}}_{k-1}\right).$$

## 3. Multi-Sensor Fusion Attitude Estimation Algorithm

**Figure 1:**
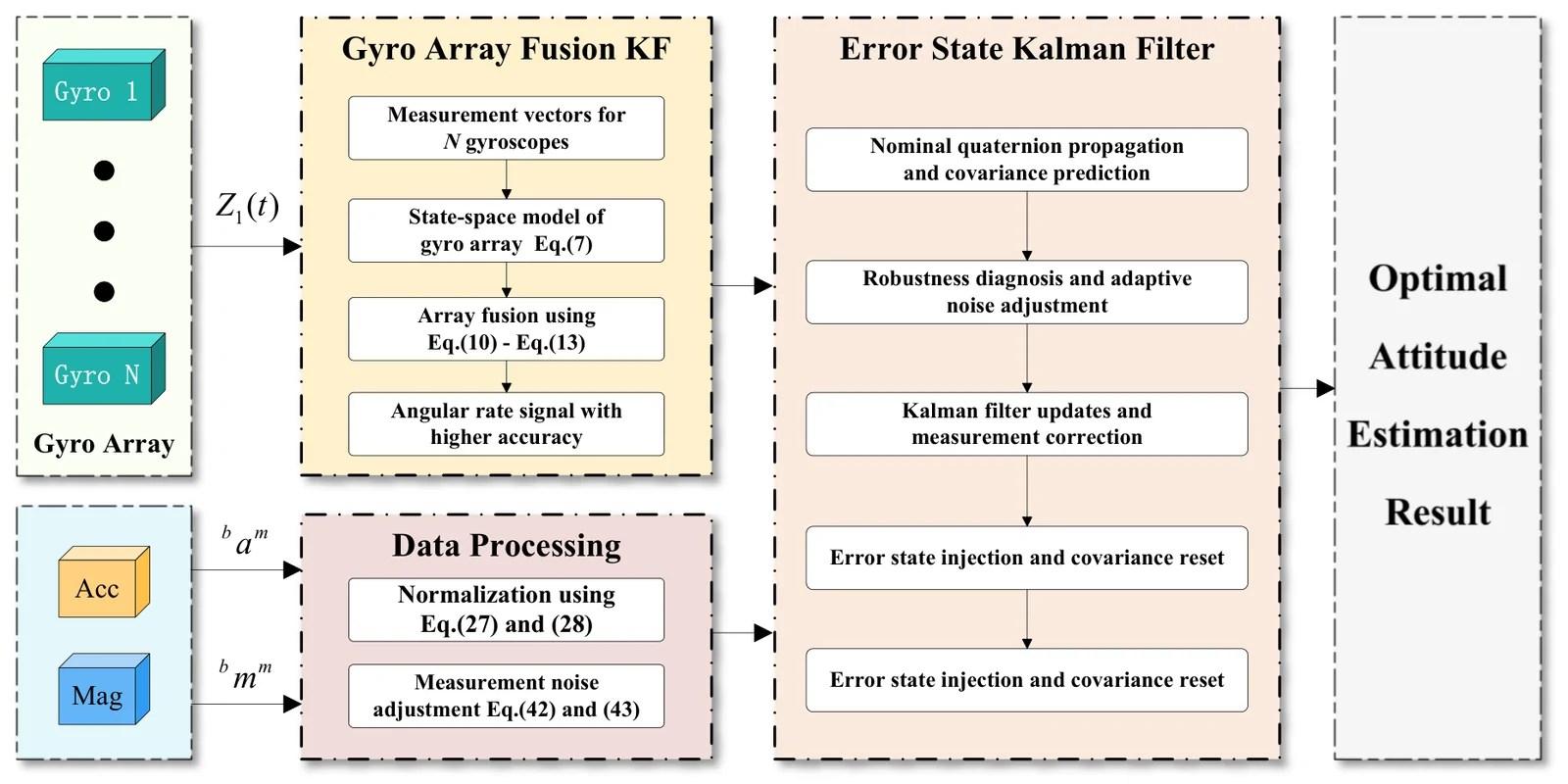
Framework of the proposed algorithm.

This section presents the design of an Error State Kalman Filter (ESKF), which integrates data from the inertial array and magnetic sensor to estimate attitude quaternions. This primarily covers the principle of strap-down attitude estimation, multi-sensor fusion, and optimal attitude quaternion Kalman filter. The overall framework diagram is shown in Figure 1.

### 3.1. Strap-Down Attitude Resolution

Select *right-front-up* as the body coordinate system *b* ($X_{b}\text{-}Y_{b}\text{-}Z_{b}$), and *north-east-up* as the geographic coordinate system *n* ($X_{n}\text{-}Y_{n}\text{-}Z_{n}$), which also serves as the navigation coordinate system. The conversion between attitude angles (yaw, pitch, roll) and quaternions is very common in the three-dimensional rotation calculation. The quaternion can be used to represent the attitude and offer high computational efficiency.

The real quaternion of the carrier is denoted as $q={\left[q_{0},q_{1},q_{2},q_{3}\right]}^{T}$. The attitude matrix $C_{b}^{n}$ represents the transformation from the body coordinate *b* to the navigation coordinate *n*:

(14) $$C_{b}^{n}=\left[\begin{array}{ccc} q_{0}^{2}+q_{1}^{2}-q_{2}^{2}-q_{3}^{2} & 2(q_{1}q_{2}-q_{0}q_{3}) & 2(q_{1}q_{3}+q_{0}q_{2})\\ 2(q_{1}q_{2}+q_{0}q_{3}) & q_{0}^{2}-q_{1}^{2}+q_{2}^{2}-q_{3}^{2} & 2(q_{2}q_{3}-q_{0}q_{1})\\ 2(q_{1}q_{3}-q_{0}q_{2}) & 2(q_{2}q_{3}+q_{0}q_{1}) & q_{3}^{2}-q_{2}^{2}-q_{1}^{2}+q_{0}^{2} \end{array}\right],$$

The derivative of the quaternion q can be expressed as:

(15) $$\dot{q}=\frac{1}{2}q⊗\left[\begin{array}{c} 0\\ \omega \end{array}\right].$$

where $\omega ={\left[\omega_{x},\omega_{y},\omega_{z}\right]}^{T}$. $C_{b}^{n}$ can be obtained by solving the quaternion differential equation, and the attitude angles can then be calculated from $C_{b}^{n}$:

(16) $$\left\{\begin{array}{c} \theta =\arcsin\left(C_{b}^{n}\left(3,2\right)\right)\\ \gamma =-\arctan\left(C_{b}^{n}\left(3,1\right)/C_{b}^{n}\left(3,3\right)\right)\\ \psi =\arctan\left(C_{b}^{n}\left(1,2\right)/C_{b}^{n}\left(2,2\right)\right) \end{array}\right.,$$

where θ is pitch angle, γ is roll angle, and ψ is yaw angle.

### 3.2. Error State Kalman Filter Algorithm Design

The Error State Kalman Filter (ESKF) decomposes the system state into a nominal state and an error state, performing linearization estimation only on the small-magnitude error state, thereby improving filtering accuracy and numerical stability. Its advantage lies in avoiding the high-order truncation errors associated with linearization in the traditional extended Kalman filter, making it particularly suitable for applications such as inertial navigation and multisensor fusion.

The system state consists of the nominal state and the error state. The nominal state is as follows:

(17) $$x_{\mathrm{nom}}=\left\{{\hat{q}}_{nb},{\hat{b}}_{g}\right\},$$

where ${\hat{q}}_{nb}$ is the nominal attitude quaternion, ${\hat{b}}_{g}$ is the estimated zero bias of the fused angular rate. The error state is represented as 6-dimensional vector, all defined in the local tangent space, and satisfy the linearity assumption:

(18) $$\delta x={\left[\delta \theta^{T}\;\delta b_{g}^{T}\right]}^{T}\in R^{6},$$

where δθ is attitude rotation error, $\delta b_{g}$ is bias error of fused gyro.

The relationship between the actual attitude and the nominal attitude is defined as:

(19) $$q_{nb}={\hat{q}}_{nb}⊗\delta q,\;\delta q={\mathrm{Exp}}_{q}\left(\delta \theta \right)\approx \left[\begin{array}{c} 1\\ \frac{1}{2}\delta \theta \end{array}\right],$$

where $q_{nb}$ represents the attitude matrix $C_{b}^{n}$, δq is the error quaternion formed by the rotation vector δθ, ${\mathrm{Exp}}_{q}\left(\cdot \right)$ is the SO(3) exponential map, which transforms a 3-dimensional rotation vector into the corresponding quaternion, ⊗ denotes quaternion multiplication.

The fused gyro measurement model satisfies the following relationship:

(20) $$\tilde{\omega }=\omega +b_{g}+n_{g},$$

where $\tilde{\omega }$ is the fused angular rate, $n_{g}$ is the Gaussian white noise.

Nominal angular rate $\hat{\omega }=\tilde{\omega }-{\hat{b}}_{g}$, substituting the error definition yields the angular rate error:

(21) $$\omega -\hat{\omega }=-\delta b_{g}-n_{g}.$$

The real and nominal quaternion dynamics are as follows:

(22) $$\dot{q}=\frac{1}{2}q⊗\left[\begin{array}{c} 0\\ \omega \end{array}\right],\;\dot{\hat{q}}=\frac{1}{2}\hat{q}⊗\left[\begin{array}{c} 0\\ \hat{\omega } \end{array}\right].$$

Take the derivative of $q=\hat{q}⊗\delta q$ and multiply both sides by ${\hat{q}}^{-1}$:

(23) $$\delta \dot{q}=\frac{1}{2}\left(\delta q⊗\omega -\hat{\omega }⊗\delta q\right).$$

Taking the small error approximation and neglecting second order terms, we can derive the differential equation for the attitude error:

(24) $$\delta \dot{\theta }=-\left[\hat{\omega }\times \right]\delta \theta -\delta b_{g}-n_{g},$$

where $\delta b_{g}=b_{g}-{\hat{b}}_{g}$, and $\dot{\delta b_{g}}=n_{bg}$, $n_{bg}$ is the Gaussian white noise, $\left[\hat{\omega }\times \right]$ is the antisymmetric matrix corresponding to true angular rate.

Thus, the error state system equation is:

(25) $$\left\{\begin{array}{c} X_{2}\left(t\right)=\delta x={\left[\delta \theta^{T}\;\delta b_{g}^{T}\right]}^{T}\\ {\dot{X}}_{2}\left(t\right)=F_{2}\cdot X_{2}\left(t\right)+G_{2}\cdot W_{2}\left(t\right) \end{array}\right.,$$

where system process noise $W_{2}={\left[n_{g}^{T},n_{bg}^{T}\right]}^{T}$, $I_{3\times 3}$ and $0_{3\times 3}$ denote identity matrix and full zero matrix, the coefficient matrices are written as:

(26) $$F_{2}=\left[\begin{array}{cc} -\left[\hat{\omega }\times \right] & -I_{3\times 3}\\ 0_{3\times 3} & 0_{3\times 3} \end{array}\right],\;G_{2}=\left[\begin{array}{cc} -I_{3\times 3} & 0_{3\times 3}\\ 0_{3\times 3} & I_{3\times 3} \end{array}\right].$$

Based on the output signals from the accelerometer and magnetometer, there are two methods for deriving the measurement equation. The core idea is to use long-term stable reference values to correct the cumulative error in attitude calculations. One approach is to transform the attitude estimation problem into a nonlinear least-squares problem and solve it using an iterative method. Another approach is to directly extract the output to establish the measurement equation. The different types of sensors in the latter method are decoupled, making it easier to handle potential disturbances.

This part utilizes a direct vector observation scheme, employing normalized acceleration and geomagnetic measurements directly as filter observations to avoid accuracy losses in intermediate steps. At the same time, a sequential update strategy is employed to achieve decoupled calibration of the two types of sensors.

Define the unit reference vectors in the navigation coordinate *n* as $r_{a}^{n}$ and $r_{m}^{n}$; $r_{a}^{n}$ is the normalized reference direction of the accelerometer, and $r_{m}^{n}$ is the normalized geomagnetic reference vector.

The outputs of the accelerometer and magnetometer, after normalization, can be expressed as

(27) za=amb∥amb∥,

(28) zm=mmb∥mmb∥.

Under the nominal attitude, the predicted observation in body coordinate *b*:

(29) $$h_{a}\left(\hat{q}\right)=C_{b}^{n}{\left(\hat{q}\right)}^{T}r_{a}^{n},$$

(30) $$h_{m}\left(\hat{q}\right)=C_{b}^{n}{\left(\hat{q}\right)}^{T}r_{m}^{n}.$$

The attitude matrix satisfies:

(31) $$C_{b}^{n}={\hat{C}}_{b}^{n}\cdot \mathrm{Exp}\left({\left[\delta \theta \right]}_{\times }\right)\approx {\hat{C}}_{b}^{n}\left(I+\left[\delta \theta \times \right]\right).$$

Thus, the observation residual can be derived as:

(32) $$r_{a}=z_{a}-h_{a}\left(\hat{q}\right)\approx \left[h_{a}\left(\hat{q}\right)\times \right]\delta \theta +v_{a},$$

(33) $$r_{m}=z_{m}-h_{m}\left(\hat{q}\right)\approx \left[h_{m}\left(\hat{q}\right)\times \right]\delta \theta +v_{m},$$

where v represents Gaussian white noise.

The Jacobian matrix can be written as:

(34) $$H_{a}=\left[\left[h_{a}\left(\tilde{q}\right)\times \right]\;0_{3\times 3}\right],$$

(35) $$H_{m}=\left[\left[h_{m}\left(\tilde{q}\right)\times \right]\;0_{3\times 3}\right].$$

For each valid observation, calculate the gain and covariance using the Kalman filter framework:

(36) $$K=P^{-}H^{T}{\left(HP^{-}H^{T}+R\right)}^{-1},$$

(37) $$P=\left(I-\mathrm{KH}\right)P^{-}{\left(I-\mathrm{KH}\right)}^{T}+{\mathrm{KRK}}^{T},$$

(38) $${\hat{X}}_{2}=K\cdot r_{i}.$$

After obtaining the error attitude estimate $\delta \hat{\theta }$, construct the correction quaternion:

(39) $$\delta \hat{q}={\mathrm{Exp}}_{q}\left(\delta \hat{\theta }\right)\approx {\left[\begin{array}{c} 1,\frac{1}{2}\delta \hat{\theta } \end{array}\right]}^{T},$$

(40) $${\hat{q}}^{+}={\hat{q}}^{-}⊗\delta \hat{q},$$

(41) $${\hat{b}}_{g}^{+}={\hat{b}}_{g}^{-}+\delta {\hat{b}}_{g}.$$

After inserting the error into the nominal state, reset the error state and covariance. The above update employs a sequential update mechanism, in which the accelerometer observation update is performed first, followed by the magnetometer observation update. Two independent 3-dimensional Kalman updates are equivalent to a single stacked 6-dimensional joint update; however, sequential updates can better handle disturbances from different sensors.

For typical sources of disturbance such as dynamic acceleration and local magnetic anomalies, smoothing and weighting are achieved by adaptively amplifying the covariance of the measurement noise. The metrics used include length deviation and direction residual.

(42) $$d_{\mathrm{norm}}=\left|\frac{∥a_{m}∥}{a_{\mathrm{ref}}}-1\right|,$$

(43) $$d_{\mathrm{angle}}=\arccos\left(z_{a}^{T}h_{a}\left(\hat{q}\right)\right),$$

where $d_{\mathrm{norm}}$ is the length deviation of accelerometer, $a_{\mathrm{ref}}$ is the reference gravity, $d_{\mathrm{angle}}$ represents the angle between the direction of the actual acceleration and the direction of the predicted gravity based on attitude. Set soft threshold $T_{\mathrm{norm}}$, $T_{\mathrm{angle}}$ and hard threshold $H_{\mathrm{norm}}$, $H_{\mathrm{angle}}$ for the two metrics, and proceed as follows in two steps:

- If $d_{\mathrm{norm}}>H_{\mathrm{norm}}$ or $d_{\mathrm{angle}}>H_{\mathrm{angle}}$, the current observation is deemed invalid, and the filter update is skipped;
- When the hard threshold is not triggered, the greater the deviation exceeds the soft threshold, the higher the noise gain: $s=\max\left(1,\frac{d_{\mathrm{norm}}}{T_{\mathrm{norm}}}\right)\cdot \max\left(1,\frac{d_{\mathrm{angle}}}{T_{\mathrm{angle}}}\right)$;
- The final adaptive measurement noise covariance is: $R_{2}=s\cdot R_{0}$, $R_{0}$ is the initial value.

The calculation method for the relevant metrics of magnetometer are the same.

## 4. Simulation and Experiment

This section presents the simulation verification and experiments for the proposed algorithm. Simulations were conducted to verify the effectiveness of the two-stage filter algorithm. Experiments were conducted to verify the actual performance in real applications.

### 4.1. Simulation Results and Analysis

To comprehensively evaluate the effectiveness of the proposed algorithm, this study constructed a navigation simulation environment. In the simulation, there are four three-axis gyroscopes, one accelerometer, and one magnetometer. Furthermore, the proposed algorithm is applicable to any reasonable number of gyroscopes.

**Figure 2:**
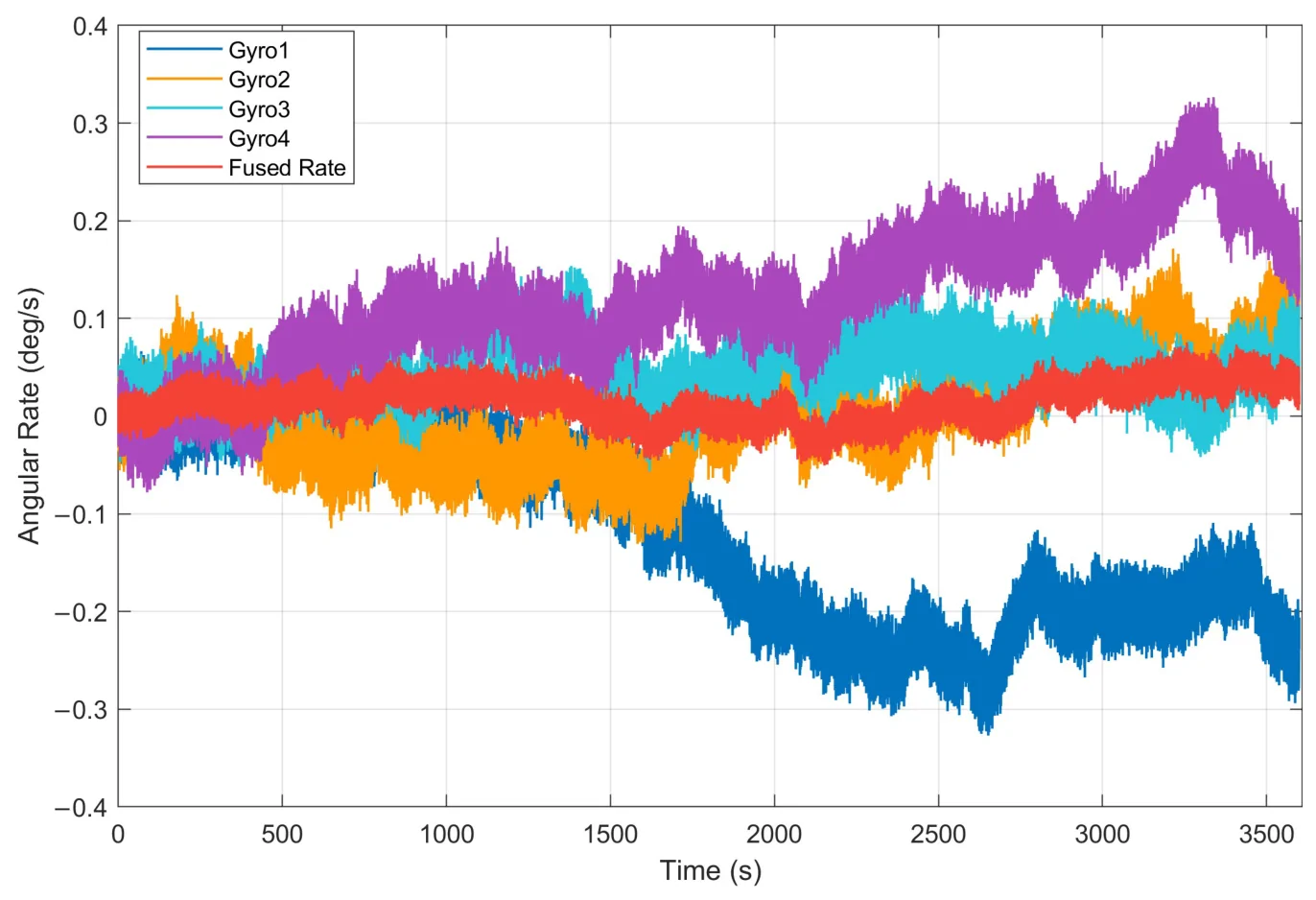
Comparison of gyro array fusion results with original angular rate.

**Figure 3:**
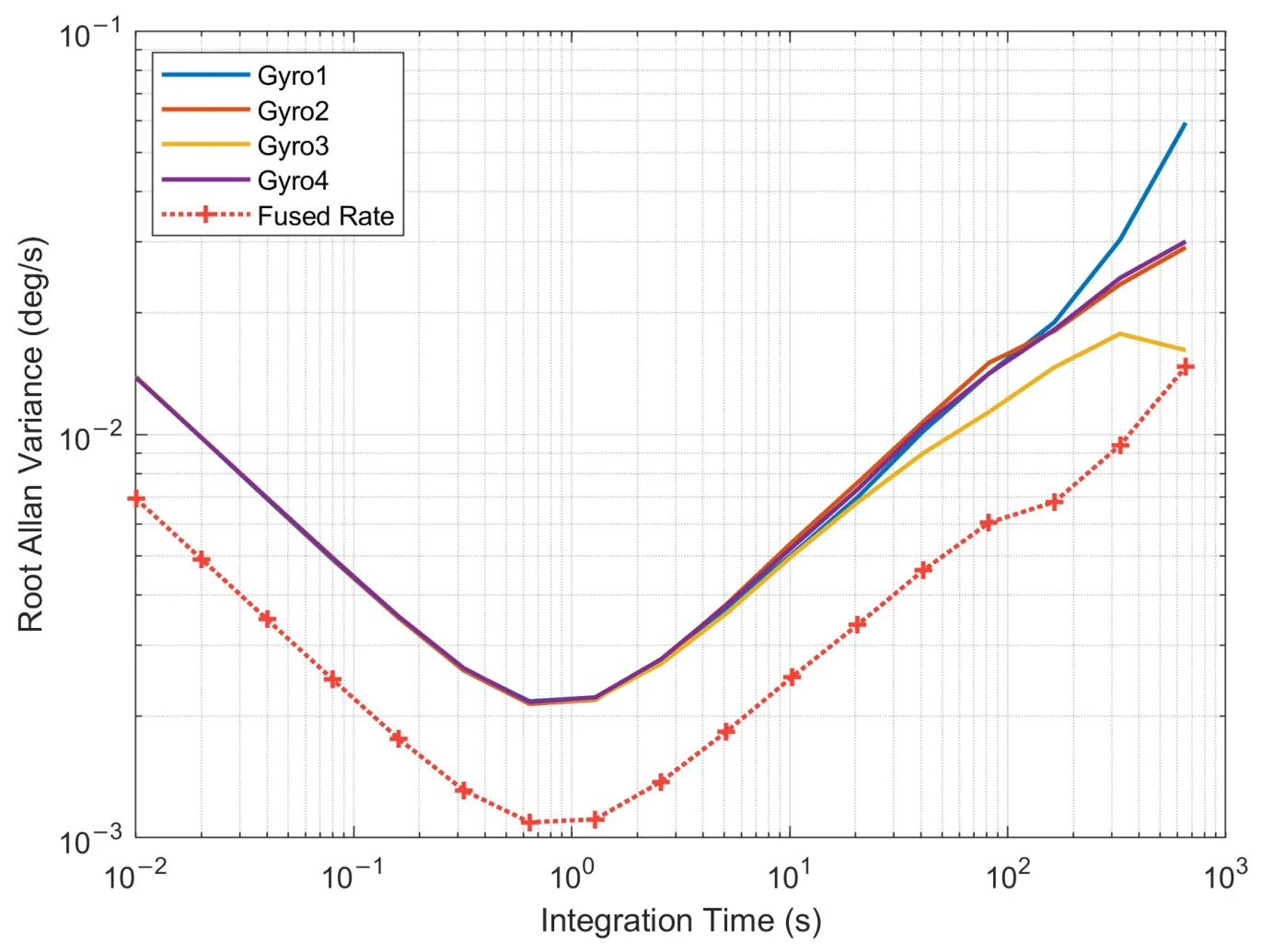
Allan variance plot under the static condition.

In array simulation, angle random walk is $0.08{}^{\circ}/\sqrt{h}$, and rate random walk is $600.0{}^{\circ}/h^{1.5}$. The performance improvement of the virtual gyroscope is verified under static conditions. The simulation results of the gyro array under static conditions are shown in Figure 2 and Figure 3.

Figure 2 indicates that the noise level of the fused angular rate is lower than that of the raw measurements, demonstrating that the Kalman filter can effectively suppress random noise. In the Allan variance plot, the curve of the fused angular rate exhibits a smaller amplitude, further confirming the reduced noise level. The ARW is reduced to $0.02{}^{\circ}/\sqrt{h}$, and the RRW is reduced to $149.83{}^{\circ}/h^{1.5}$. Furthermore, the simulation results demonstrate that employing the redundant array can effectively mitigate subsequent error accumulation.

For the second-stage attitude estimation algorithm, simulation in dynamic scenarios was used to verify the algorithm’s effectiveness. Considering the motion characteristics of the actual vehicle during the cruise phase, we designed a composite motion trajectory with low dynamics and a long period. In addition, to verify the robustness of the algorithm against external disturbances, dynamic acceleration and magnetic disturbance were introduced.

**Figure 4:**
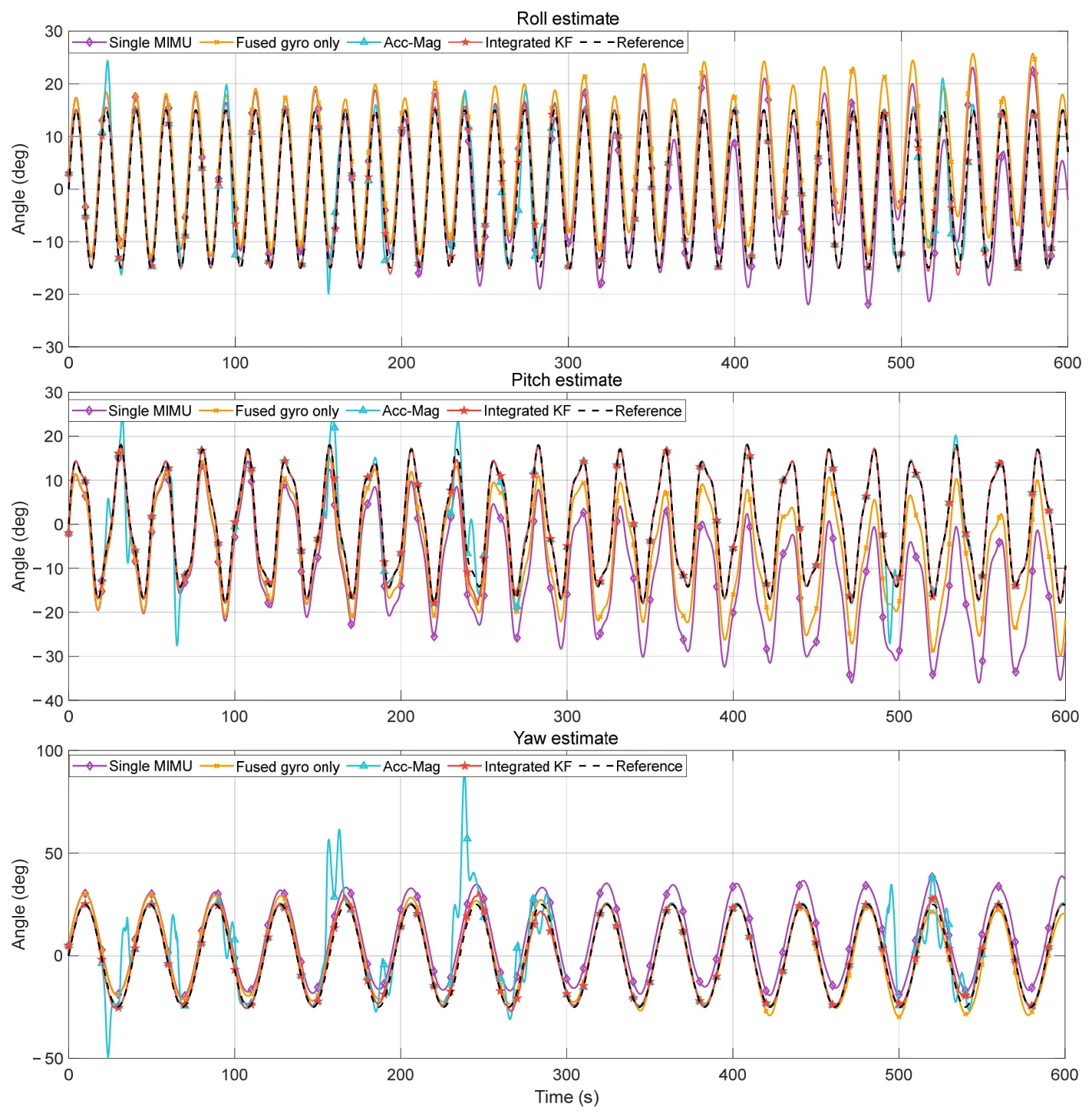
Comparison of the reference value and attitude estimation results using different methods in simulation.

**Figure 5:**
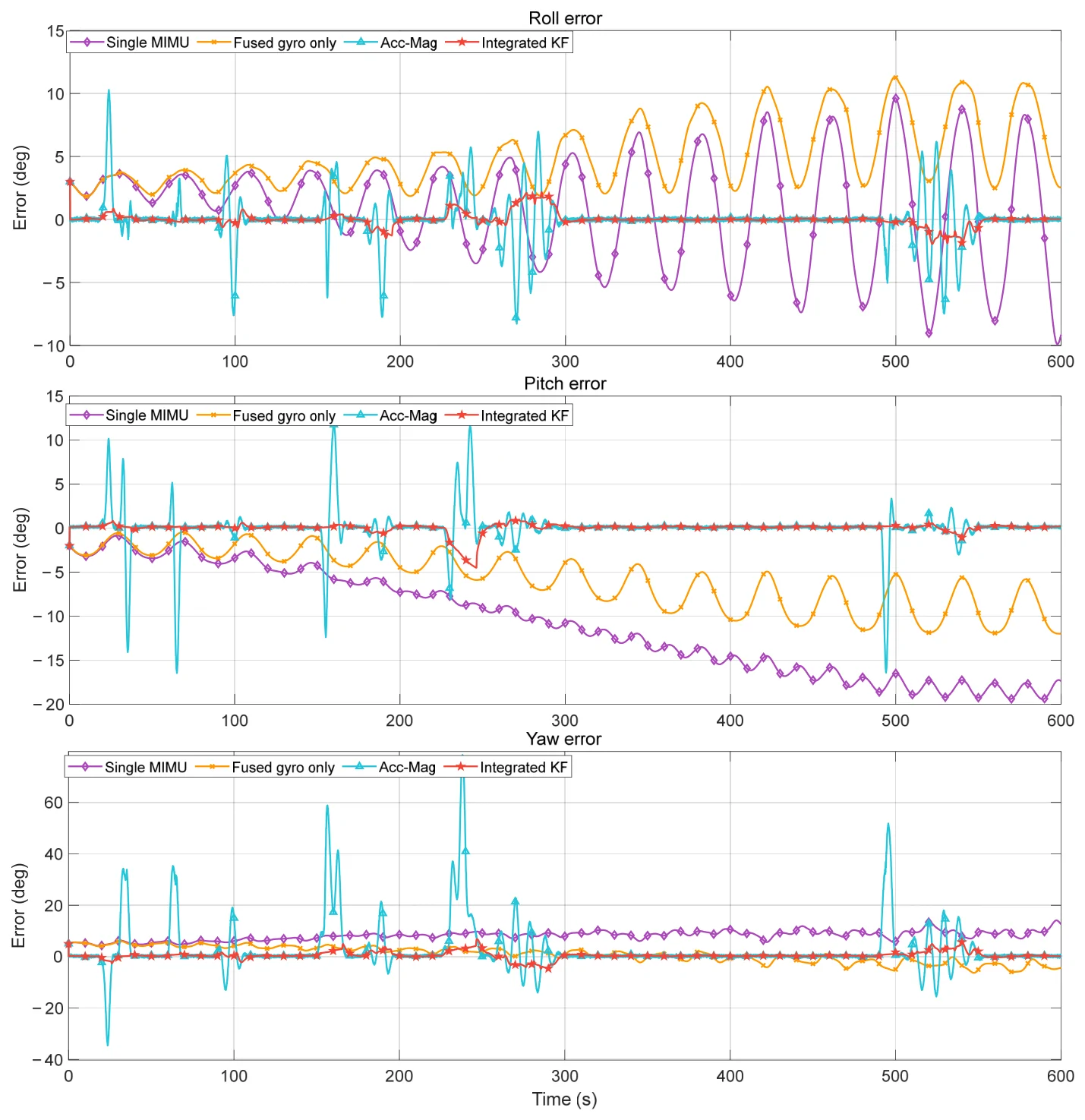
Comparison of attitude estimation errors.

The simulation results of the ESKF algorithm are shown in Figure 4 and Figure 5. Single MIMU refers to the strapdown attitude solution from a single gyroscope. The fused gyro uses the fused angular rate signal. Acc-Mag represents the attitude estimate obtained through the iterative solution method using the accelerometer and the magnetometer [22]. The proposed integrated attitude estimation algorithm achieves the highest accuracy. In contrast, relying solely on gyroscope measurements leads to rapid divergence of the attitude error. Meanwhile, fusing the output signals of the four gyroscopes improves the measurement accuracy of the angular rate. However, the accumulation of errors in strapdown attitude computation still exists.

Additionally, the attitude of the carrier can be determined using the measurements from the accelerometer and the magnetometer. However, this method is highly susceptible to disturbance. As shown in Figure 4, both dynamic acceleration and magnetic field disturbances can lead to severe attitude errors. Conversely, the ESKF can adaptively adjust the noise covariance in the presence of disturbances, and the sequential update method decouples the accelerometer and magnetometer. The accelerometer primarily constrains roll and pitch errors, while the magnetometer mainly constrains the heading angle error.

The integrated algorithm combines the advantages of these two approaches. By using gyro integration results as a basis and introducing the invariant external references, it resolves the problem of error accumulation and yields high-precision attitude angles. The adaptive noise covariance used also helps suppress the effects of external disturbances.

### 4.2. Experimental Results and Analysis

**Figure 6:**
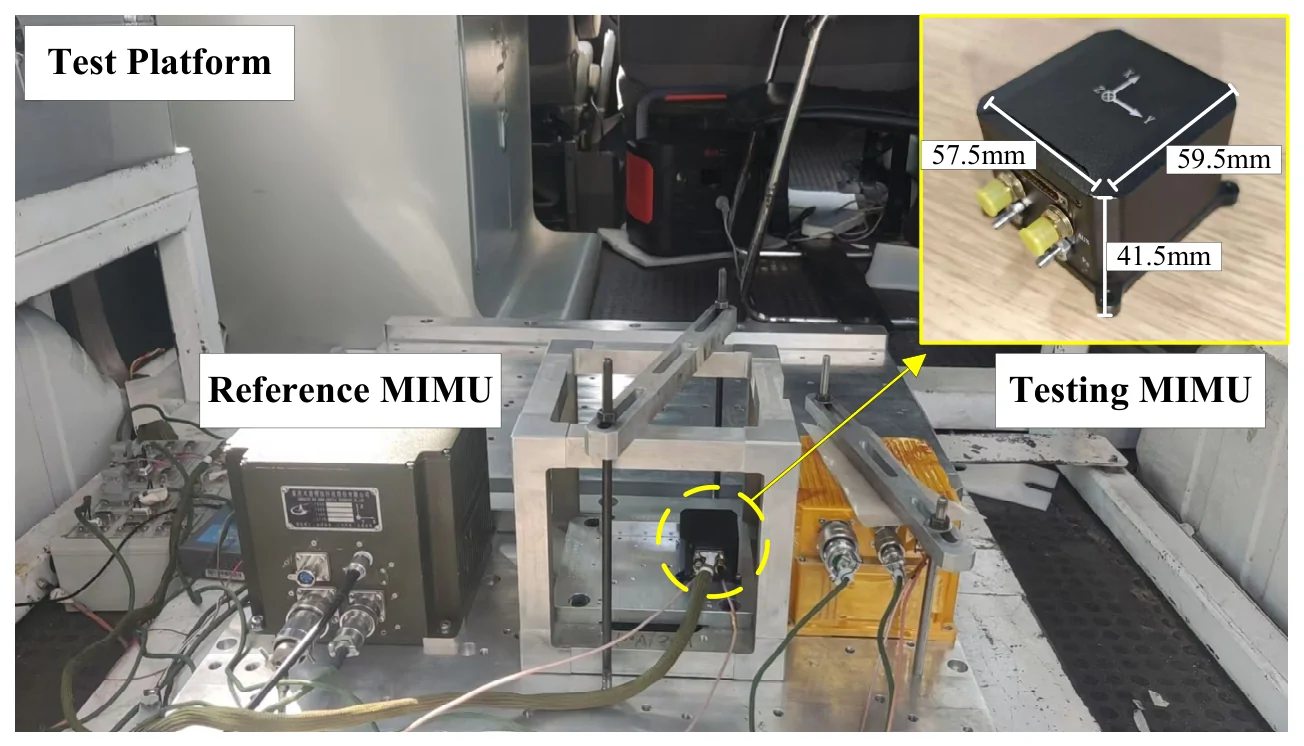
The designed multi-sensor testing MIMU and the experimental platform for field tests.

To further validate the effectiveness of the proposed algorithm in actual environments, field tests were designed. The test platform is shown in Figure 6.

The integrated navigation system consists of a micro-inertial measurement unit and a magnetometer and can measure navigation data along three axes. The micro-inertial measurement unit includes eight gyroscopes and one accelerometer. The gyroscopes are arranged in a planar array. A high-precision fiber-optic inertial measurement unit (FOG-IMU) was used as the reference system. The experiment is divided into two parts: gyro array fusion and attitude estimation.

#### 4.2.1. Experimental Results for Gyroscope Array Fusion

**Figure 7:**
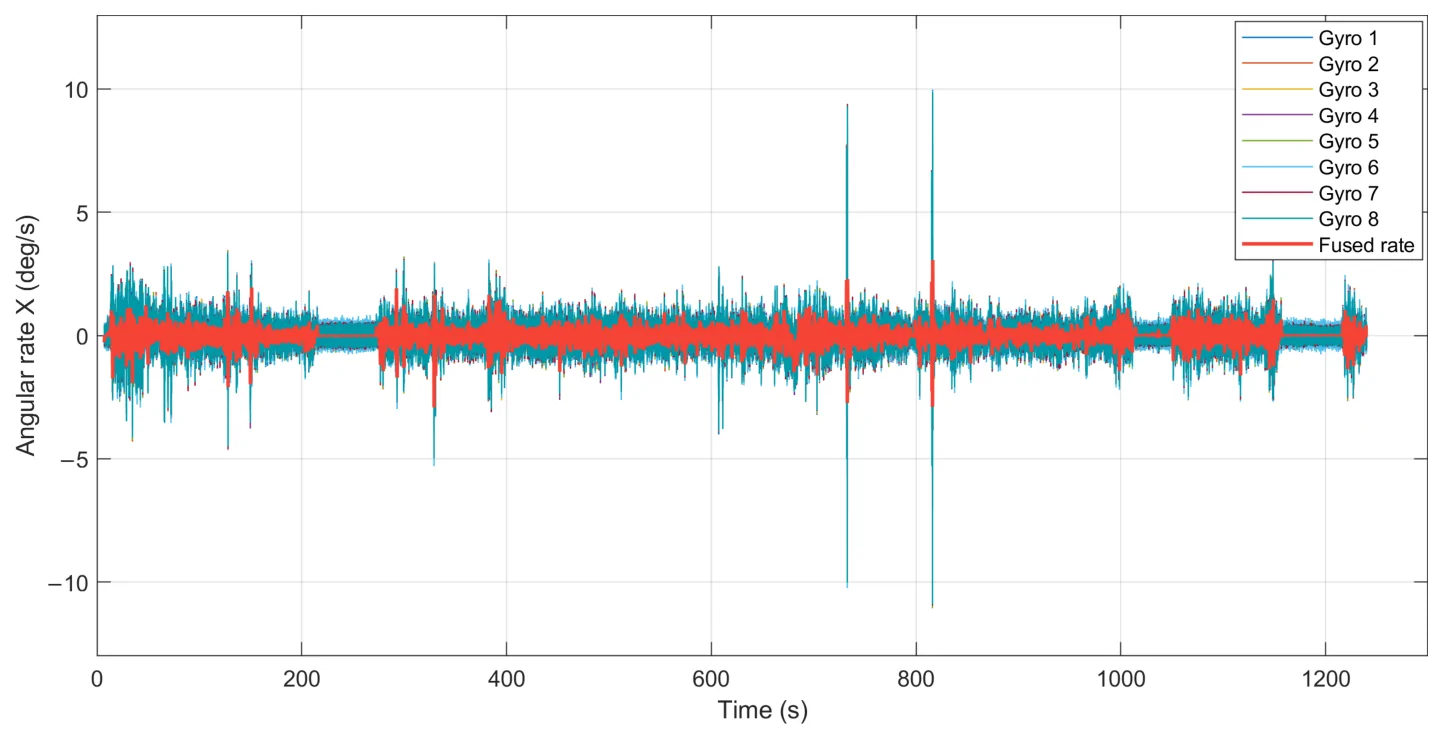
The original angular rate and the fused angular rate along the X-axis.

**Figure 8:**
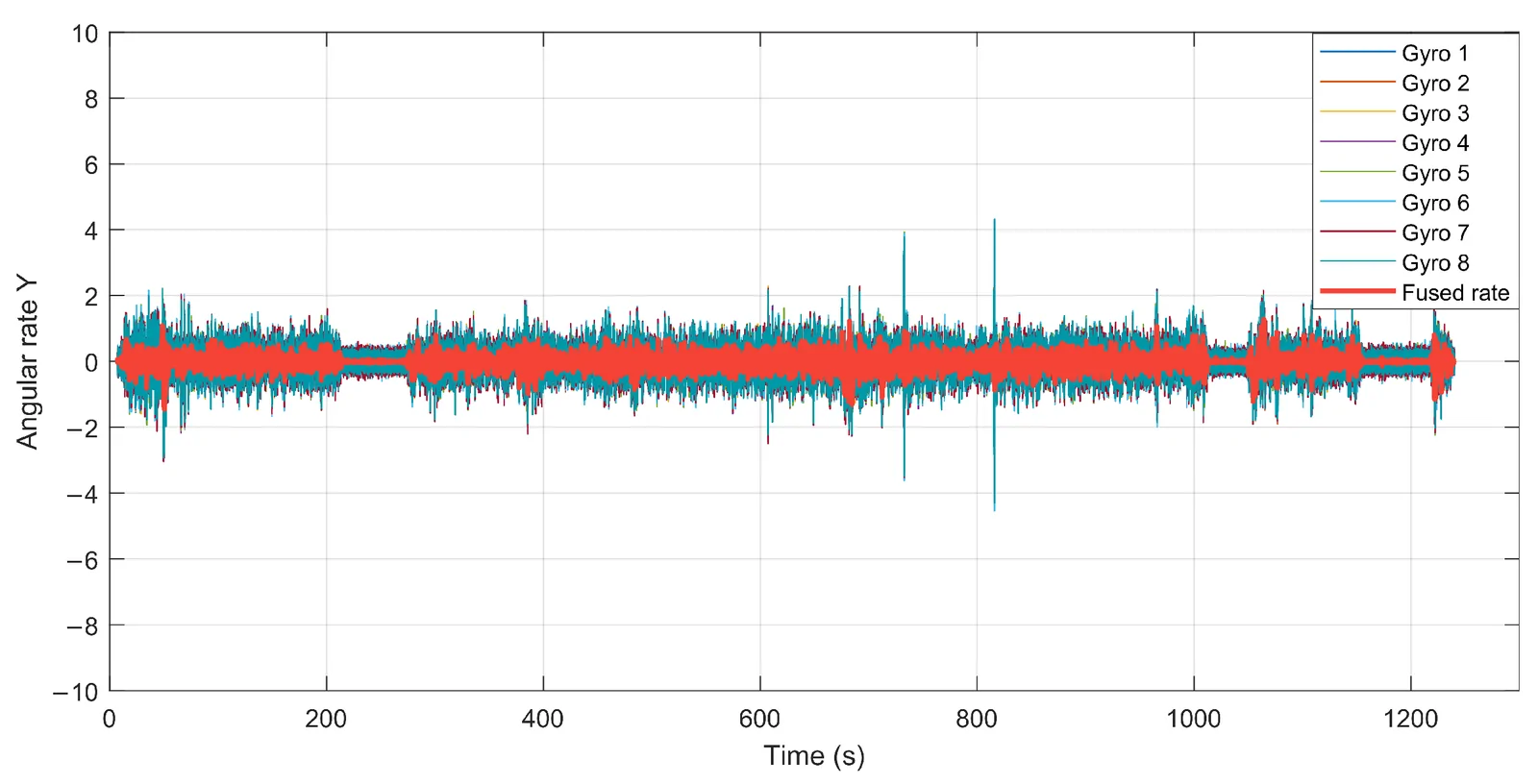
The original angular rate and the fused angular rate along the Y-axis.

**Figure 9:**
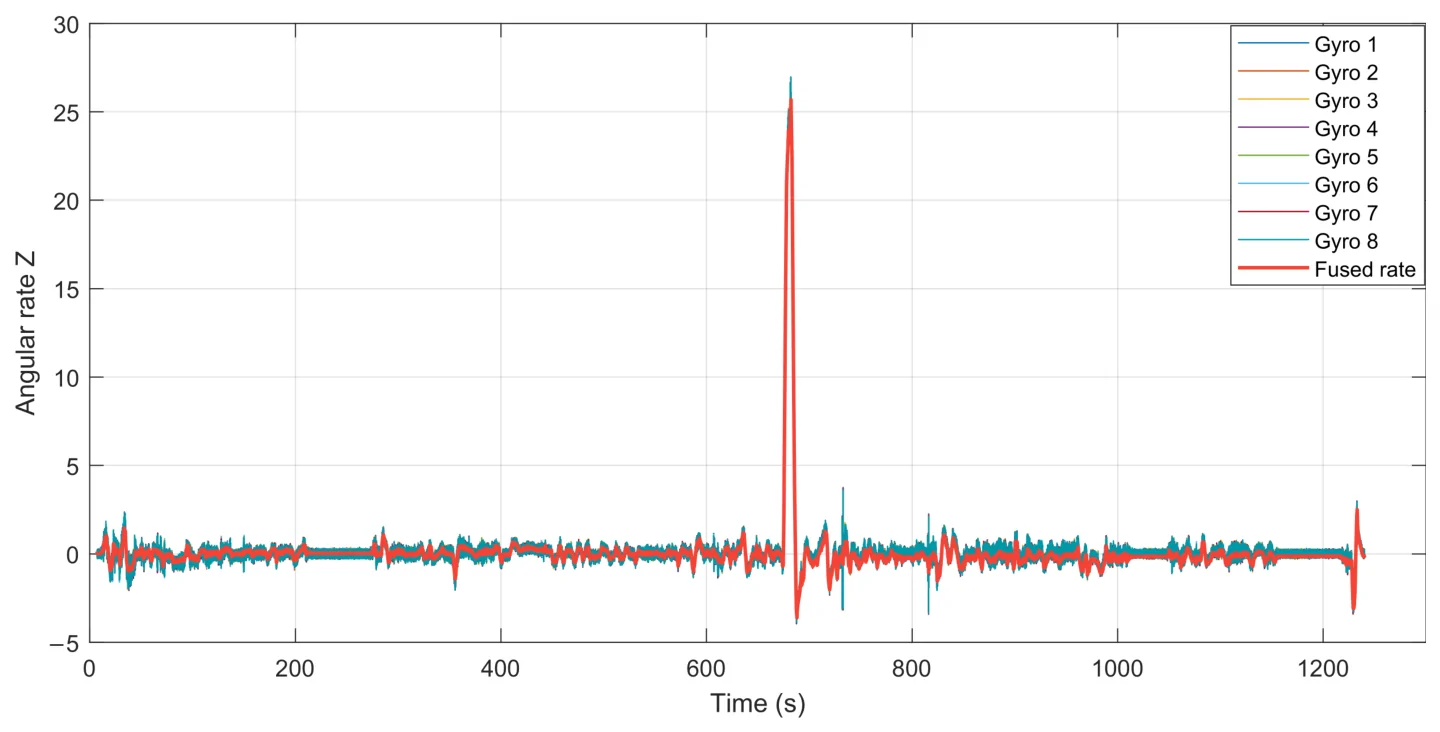
The original angular rate and the fused angular rate along the Z-axis.

The test platform is equipped with a MEMS gyro array consisting of eight homogeneous three-axis gyroscopes, capable of outputting three-axis measurement signals. In Section 2, the Kalman filter algorithm for fusing redundant measurements is designed to serve as the first-stage filter in the proposed integrated navigation algorithm. The angular rate fusion results are shown in Figure 7, Figure 8 and Figure 9.

The test results show that the designed Kalman filter algorithm can effectively improve the accuracy of angular rate measurements. Under experimental conditions, the impact of random noise on attitude estimation was reduced. The first-stage filtering removes random noise from the angular rate signal before it is used. As in the simulation, the accuracy of the solution obtained using a fused rate is much higher than that obtained using a single gyroscope.

Array fusion is the first-stage filtering process designed to provide more accurate angular rate for the second-stage attitude estimation. After completing the fusion of redundant gyroscopes, the strapdown attitude calculation can be performed to obtain a preliminary estimate of the attitude quaternion.

#### 4.2.2. Experimental Results for Attitude Estimation

The navigation simulation validated the feasibility of the proposed algorithm. To further verify the performance of the algorithm in a real-world environment, two sets of field tests were designed. The experiments adopt the TY-MIGA-100A integrated navigation system. This system supports static and dynamic initial alignment and compensates for installation errors via external parameters. It can also perform magnetometer calibration and compensate for the hard-iron and soft-iron effects. Calibration was completed using the manufacturer-provided software, which is not elaborated here since it is beyond the scope of this work.

The parameters used in the navigation experiments are shown in Table 1. Set the sampling frequency to 100 Hz. The filter is initialized via the static initialization method, where the initial attitude is calculated using accelerometer and magnetometer measurements. Part of the noise parameters are taken from the device datasheet, while the rest are obtained by performing Allan Variance analysis on measured static-state data. The hard threshold indicates that data are invalid when sensor deviations exceed the threshold. The soft threshold means deviations are tolerable and only require adjustment of the noise covariance.

Various methods are used to estimate attitude, including strapdown inertial integration, estimation using accelerometer and magnetometer (Acc-Mag), and the proposed error state Kalman filter (ESKF). The results are shown in Figure 10, Figure 11, Figure 12, Figure 13, Figure 14, Figure 15, Figure 16, Figure 17, Figure 18, Figure 19, Figure 20 and Figure 21. Table 2 and Table 3 present the Root Mean Square Error (RMSE) and Mean Absolute Error (MAE) metrics for the two tests.

(44) $$\mathrm{RMSE}=\sqrt{\frac{1}{n}\sum_{i=1}^{n}{\left(Euler_{E}-Euler_{R}\right)}_{k}^{2}}$$

(45) $$\mathrm{MAE}=\frac{1}{n}\sum_{k=1}^{n}\left|{\left({\mathrm{Euler}}_{E}-{\mathrm{Euler}}_{R}\right)}_{k}\right|$$

where ${\mathrm{Euler}}_{E}$ is the estimated value, ${\mathrm{Euler}}_{R}$ is the reference value.

**Figure 10:**
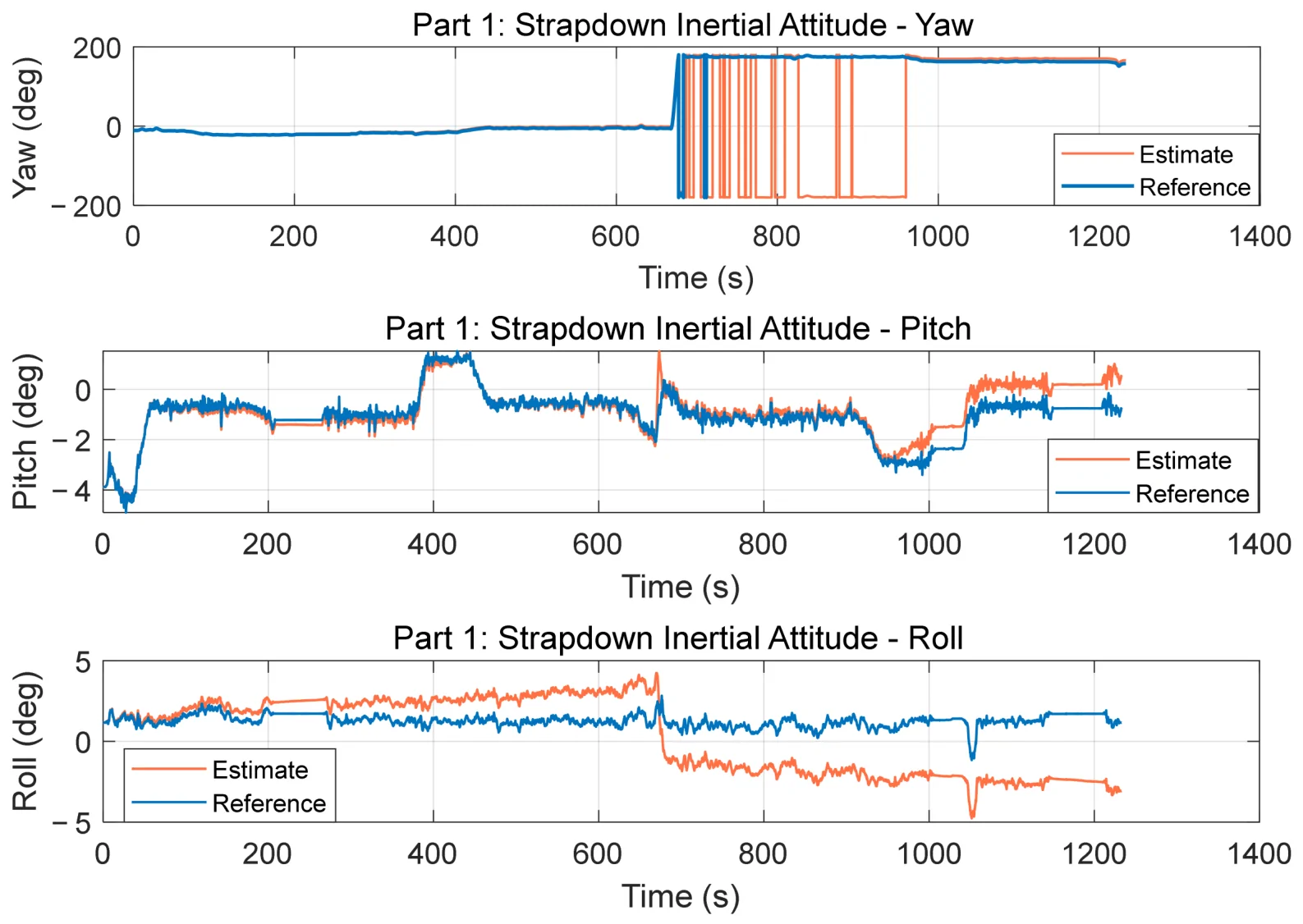
The reference value and strapdown attitude estimation results for Test 1.

**Figure 11:**
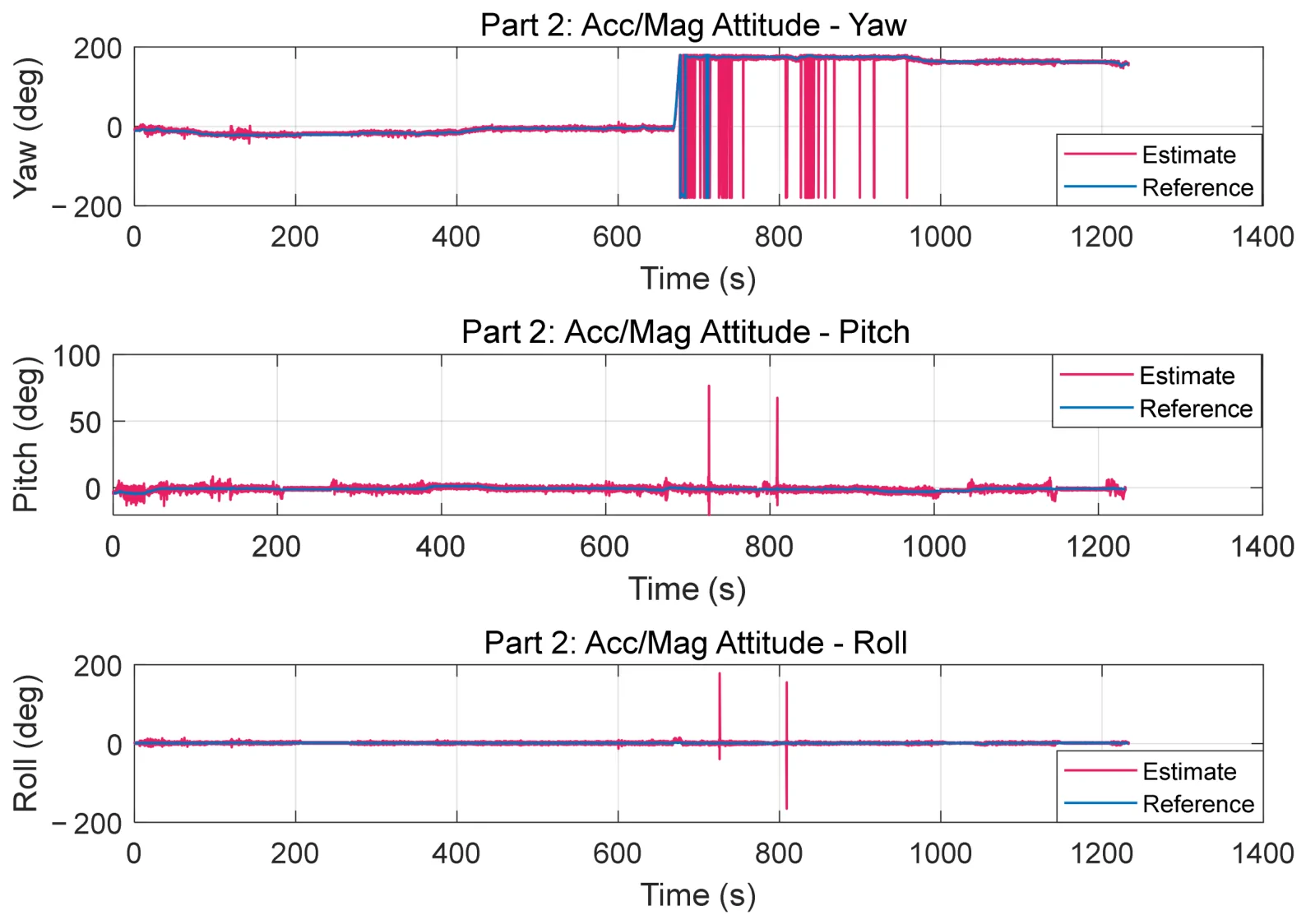
The reference value and attitude estimation results determined using the accelerometer and magnetometer in Test 1.

**Figure 12:**
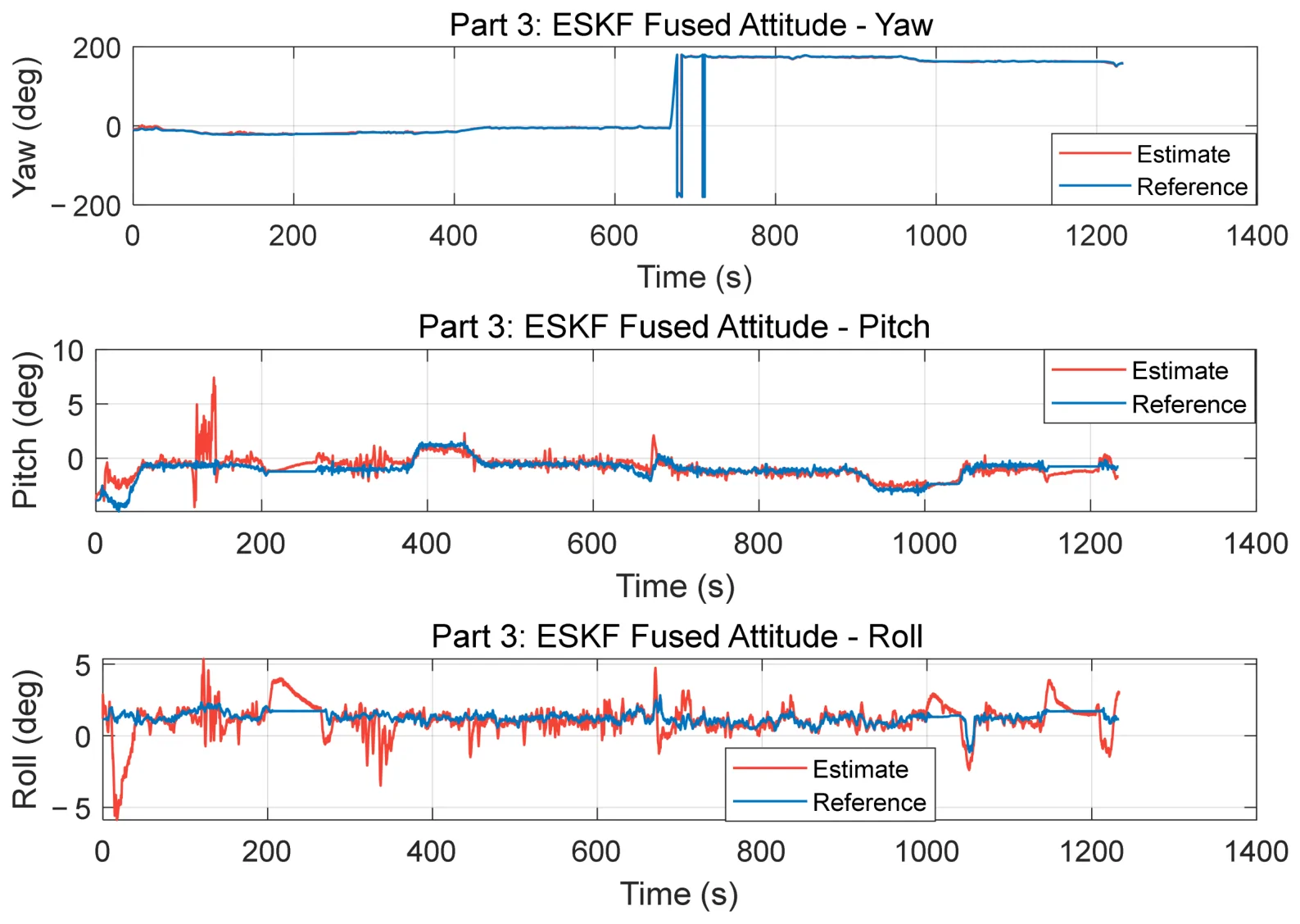
The reference value and attitude estimation results obtained using error state Kalman filter in Test 1.

**Figure 13:**
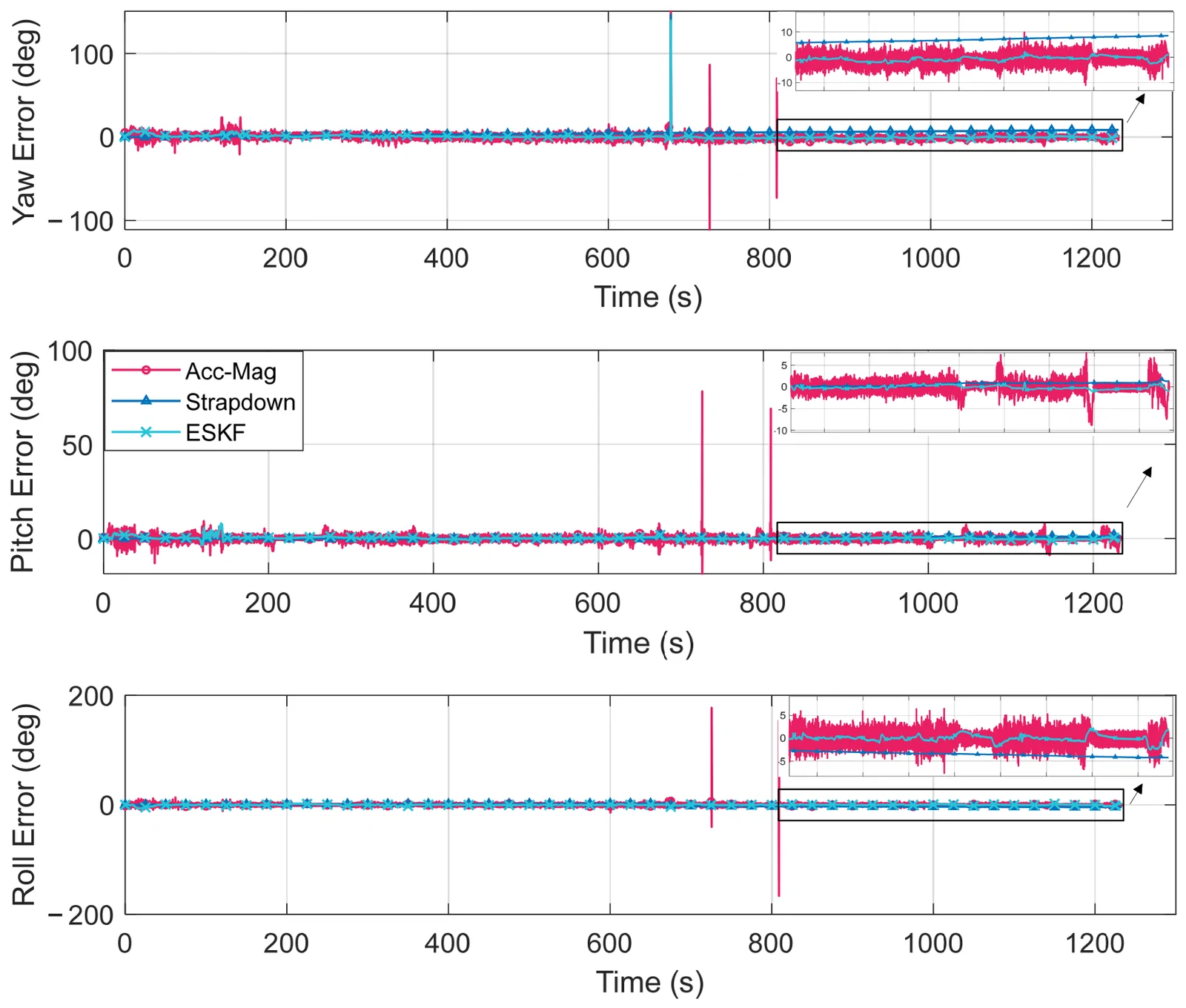
Comparison of attitude estimation errors determined via different methods in Test 1.

The attitude angle estimation results for Test 1 are shown in Figure 10, Figure 11, Figure 12 and Figure 13. As can be seen from the results, all three methods are capable of estimating the attitude of carrier.

The results of strapdown inertial integration are shown in Figure 10. The estimated attitude angles are relatively smooth, but errors accumulate over time. In Figure 11, there is no error accumulation when determining attitude using the accelerometer and magnetometer. However, due to the external disturbances, high frequency fluctuations are present in the results. Figure 12 shows that the proposed error state Kalman filter can achieve accurate estimation of attitude angles. Furthermore, the estimation results do not exhibit the error accumulation shown in Figure 10, and the issue of low accuracy shown in Figure 11 has been resolved. The error comparison in Figure 13 shows that the ESKF achieves the highest accuracy.

**Figure 14:**
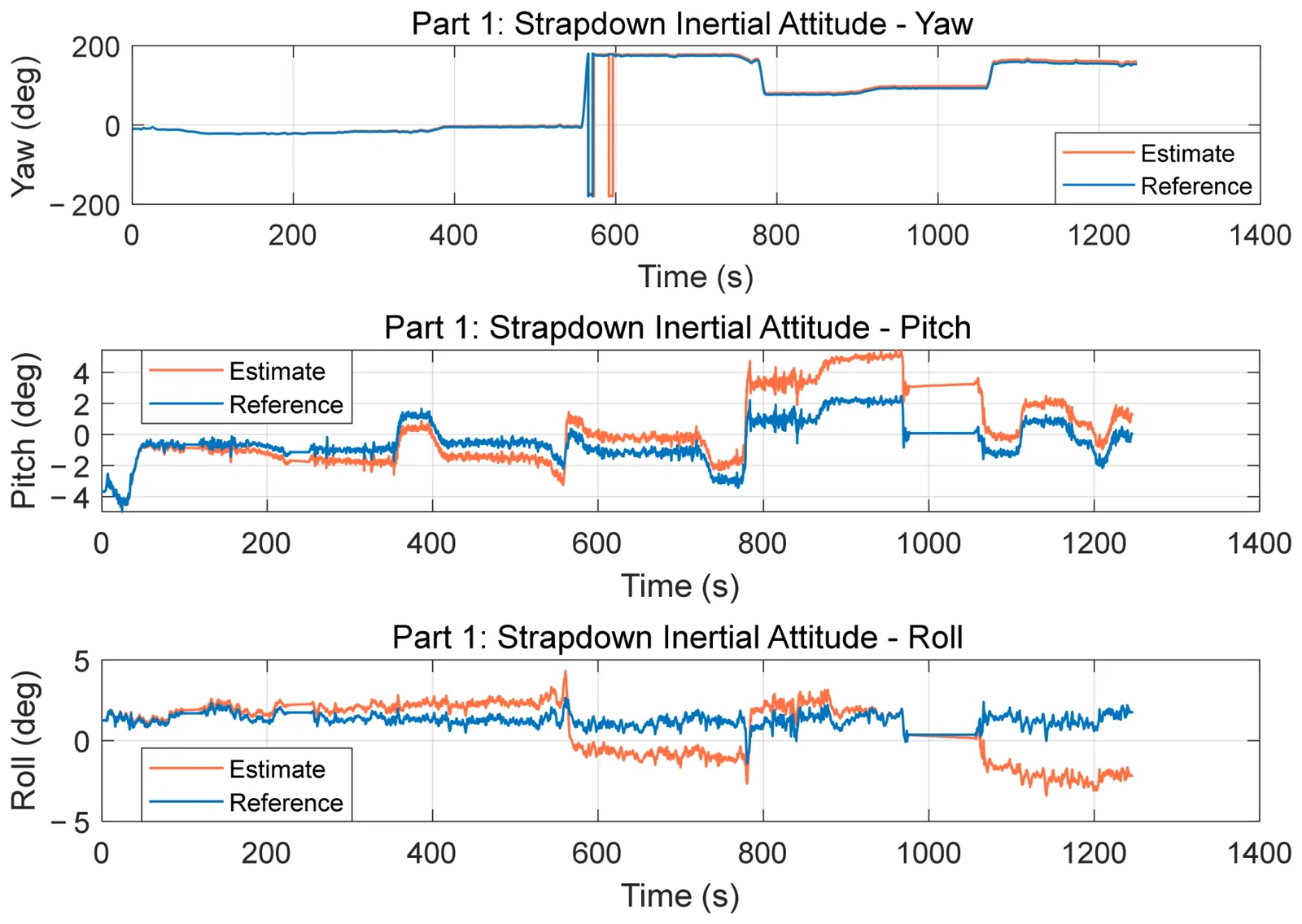
The reference value and strapdown attitude estimation results for Test 2.

**Figure 15:**
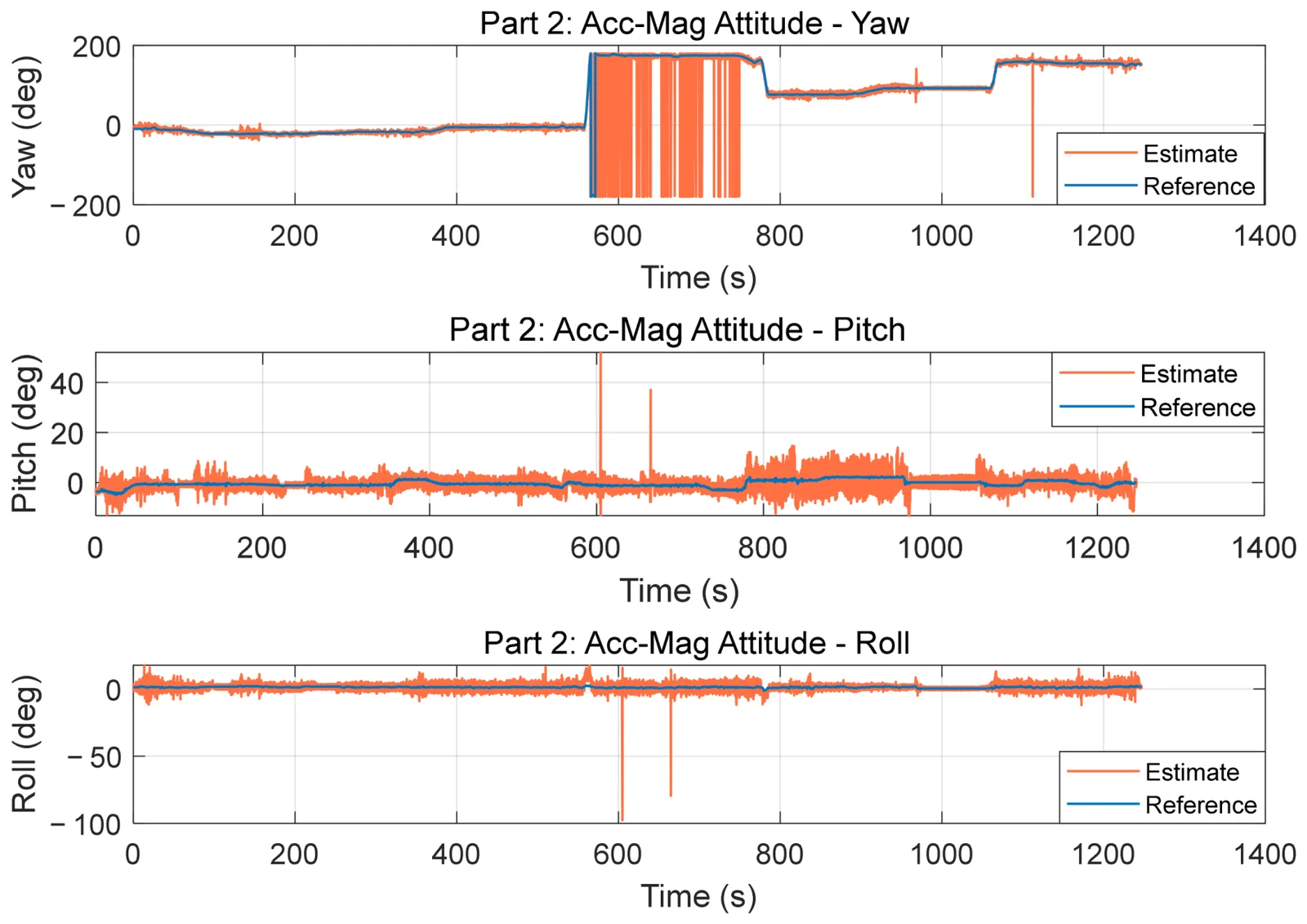
The reference value and attitude estimation results determined using the accelerometer and magnetometer in Test 2.

**Figure 16:**
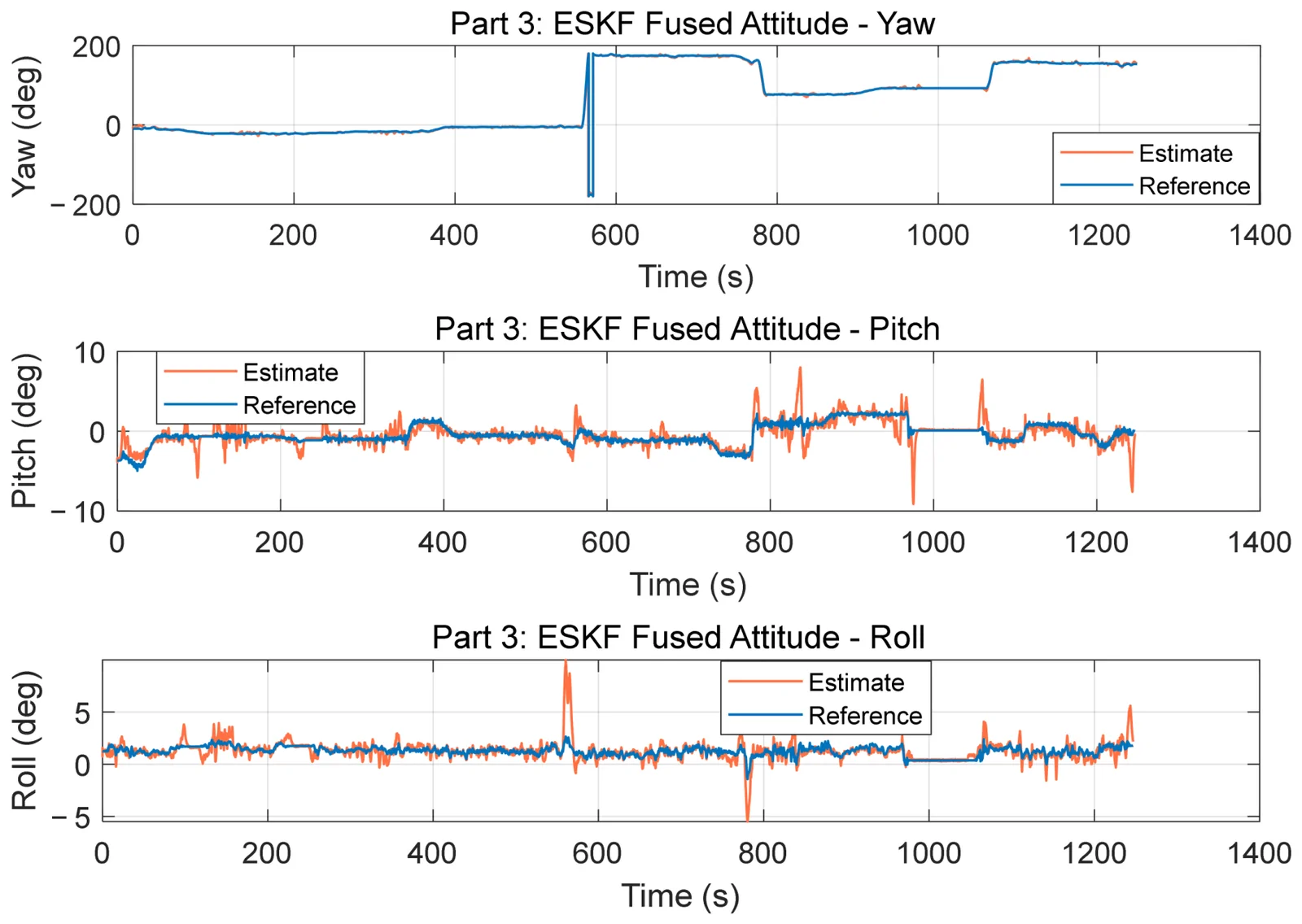
The reference value and attitude estimation results obtained using error state Kalman filter in Test 2.

**Figure 17:**
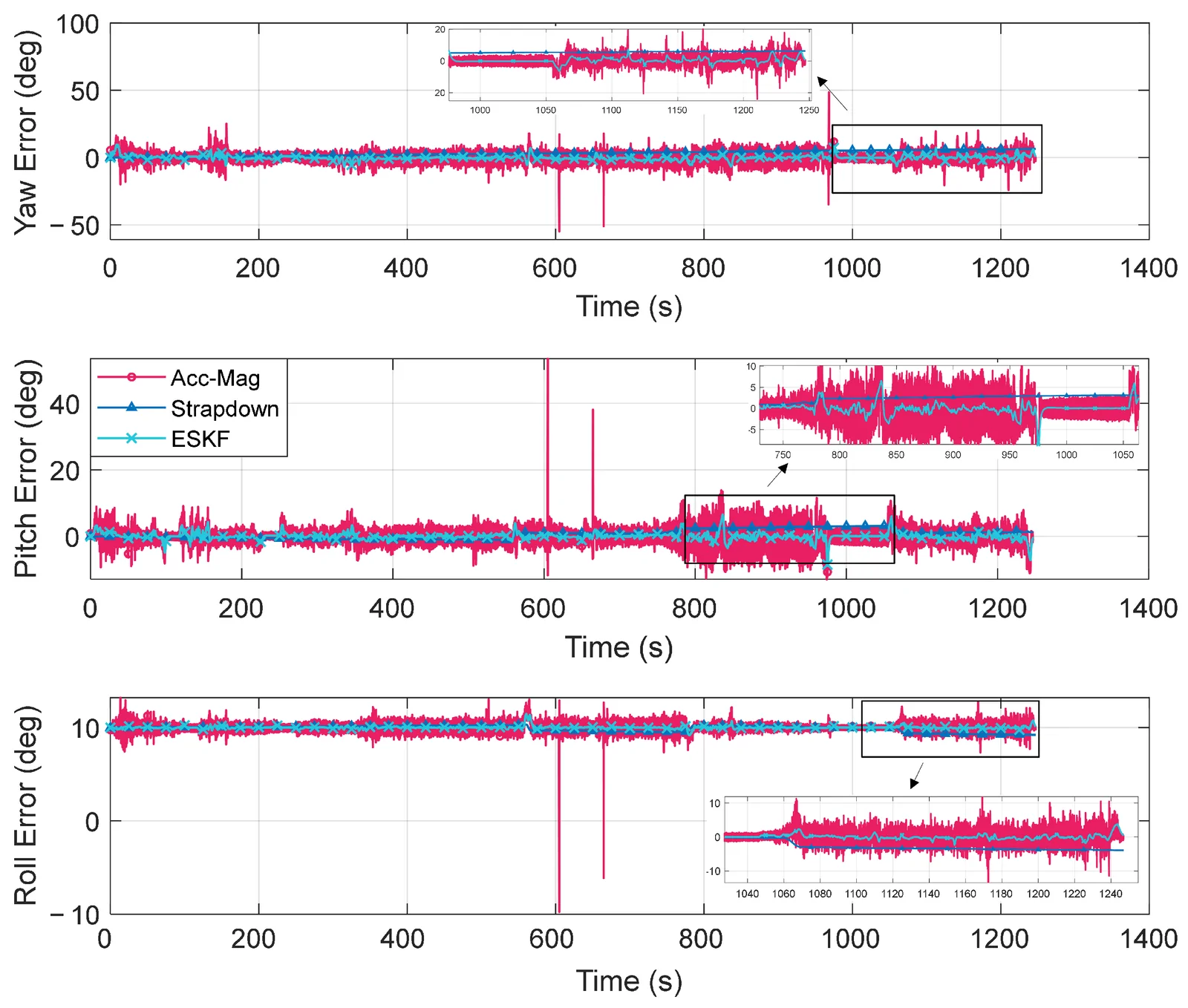
Comparison of attitude errors determined via different methods in Test 2.

To further validate the performance of the ESKF algorithm, a different vehicle route was selected for test 2. The experimental results are shown in Figure 14, Figure 15, Figure 16 and Figure 17.

From Figure 14, Figure 15, Figure 16 and Figure 17, the experimental results lead to the same conclusion in Test 2. The proposed error state Kalman filter combines the advantages of both methods, addresses the issues of error accumulation and high-frequency disturbances, and yields the optimal attitude estimation. As shown in Table 2 and Table 3, the RMSE and MAE values for the ESKF are significantly lower than those of other comparison methods.

**Figure 18:**
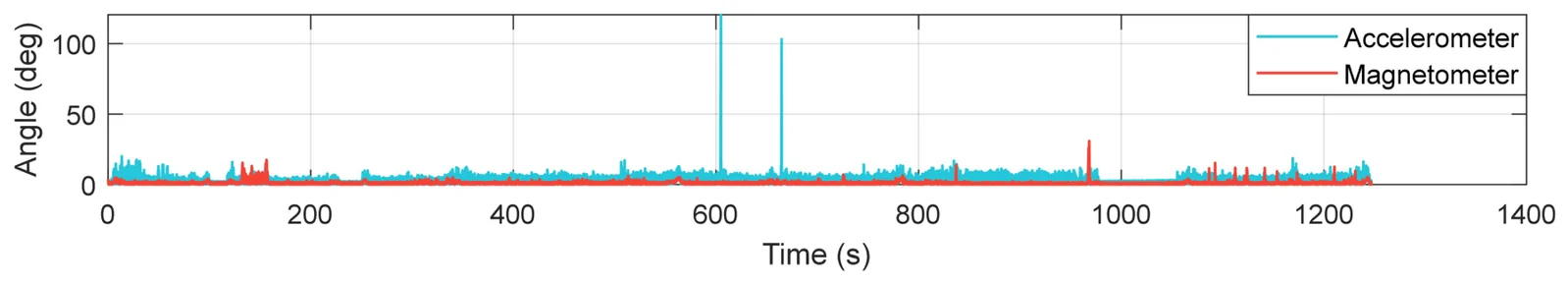
The observation residual angle in Test 2.

**Figure 19:**
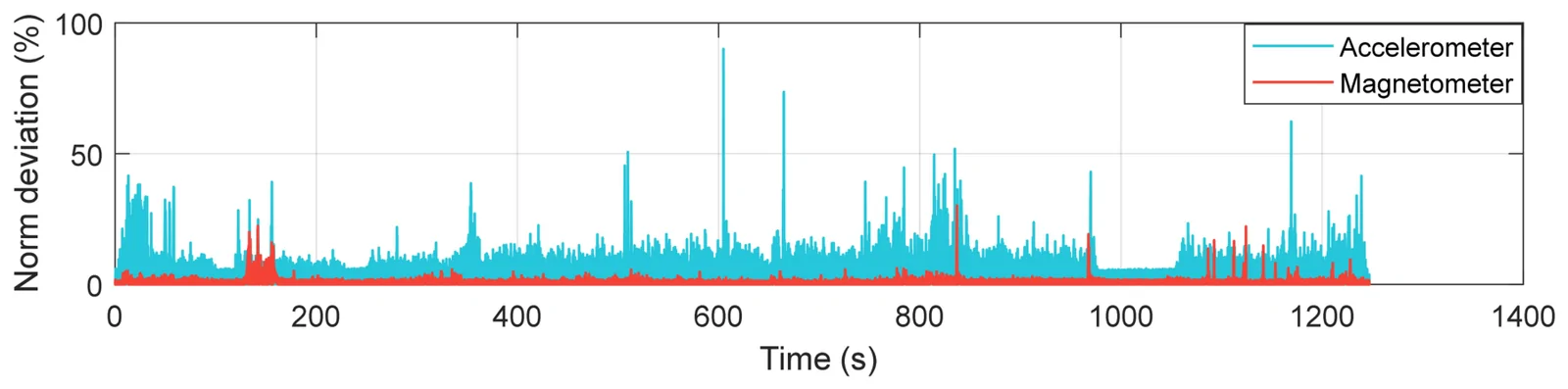
The norm deviation percentage in Test 2.

**Figure 20:**
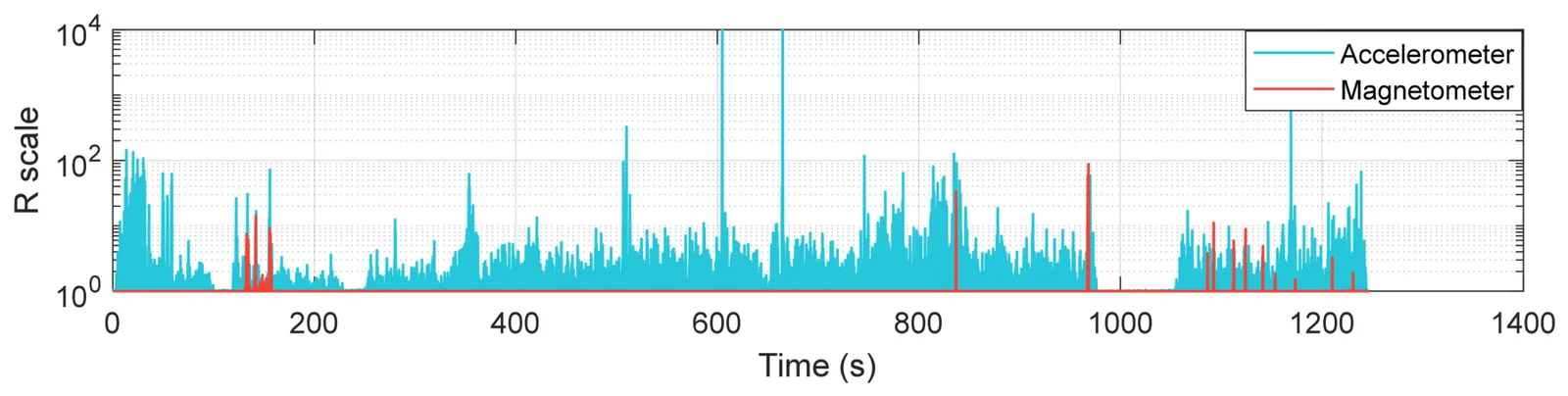
The noise covariance scaling factor in Test 2.

**Figure 21:**
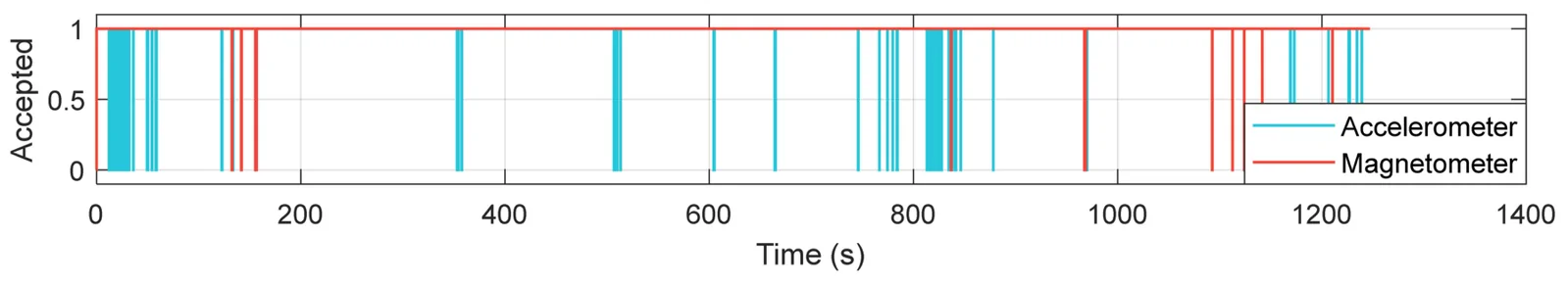
The observation acceptance flag in Test 2.

To verify the effectiveness of the disturbance handling mechanism used in the proposed algorithm, a more detailed analysis of the disturbance is provided using Test 2 as an example. The results are shown in Figure 18, Figure 19, Figure 20 and Figure 21.

Figure 18 and Figure 19 show observation residual angle and norm deviation percentage in the experiment. Observation residual angle and norm deviation represent the errors between the actual measurements from component sensors and the theoretical calculations. It shows that a strong acceleration disturbance exists in the experiment, while the overall magnetic field deviation is low, indicating that the magnetic environment is relatively stable. Figure 20 represents the noise covariance scaling factor. When observation anomalies are detected, the observation noise covariance is inflated to down-weight the anomalous measurements, with a larger scaling factor reflecting lower reliability of the corresponding sensor data. Figure 21 represents the observation acceptance flag, which indicates that the mechanism ensures system stability and robustness by rejecting anomalous observations. The analysis results show that the disturbance handling mechanism can improve the robustness of proposed algorithm.

## 5. Conclusions

In this work, a multi-sensor fusion attitude estimation algorithm is proposed. The integrated system includes a redundant array of homogeneous MEMS gyroscopes, as well as an accelerometer and a magnetometer. A two-stage data fusion algorithm was employed. The first-stage Kalman filter is used to fuse angular rate signals from multiple gyroscopes. In the second stage, the error state Kalman filter receives measurements from the accelerometer and magnetometer, updates the error state, and produces the final attitude estimate after correction. Simulation and experimental results show that the gyro array can effectively improve the measurement accuracy of angular rate and reduce the impact of random noise. Meanwhile, the proposed error state Kalman filter can obtain the optimal attitude estimate. The error profile of ESKF is much smoother and with lower values. Compared to the RMSE of strapdown inertial solution, Roll was reduced by 55.34%, Pitch by 30.25%, and Yaw by 55.71%. The proposed method offers an effective approach to low-cost, high-precision attitude estimation. In the future, further research can be conducted to expand the number of array sensors, improve the noise adaptive mechanism, and extend the application scenarios, while the current experimental validation is limited, a more comprehensive comparison with other advanced algorithms is also identified as a primary direction in our future work.

## Figures and Tables

**Table 1:** Parameter setting.

Parameter	Value
Sampling Frequency	100 Hz
Initial Attitude Error Std	5°
Initial Bias Error Std	1°/s
Acc Noise Std	0.05
Acc Bias Stability	0.01 g
Gyro Noise Std	$0.2{}^{\circ}/\sqrt{h}$
Gyro Bias Stability	10°/h
Mag Noise Std	0.04
Correlation Time Constant	1000 s
Acc Soft/Hard Norm Threshold	0.05/0.25
Mag Soft/Hard Norm Threshold	0.08/0.30
Acc Soft/Hard Angle Threshold	10°/60°
Mag Soft/Hard Angle Threshold	12°/45°

**Table 2:** Comparison of statistical metrics for Test 1.

Metrics	Euler Angle	Strapdown	Acc-Mag	ESKF
RMSE (deg)	Roll	2.4036	1.8734	1.0353
	Pitch	0.7692	1.4077	0.4587
	Yaw	4.9495	2.7002	1.5927
MAE (deg)	Roll	2.0612	1.2288	0.6056
	Pitch	0.4748	0.9226	0.3021
	Yaw	4.3112	1.9626	1.0787

**Table 3:** Comparison of statistical metrics for Test 2.

Metrics	Euler Angle	Strapdown	Acc-Mag	ESKF
RMSE (deg)	Roll	1.6566	1.8786	0.7399
	Pitch	1.5415	2.1441	1.0752
	Yaw	3.7437	3.4635	1.6579
MAE (deg)	Roll	1.2369	1.3331	0.3949
	Pitch	1.2517	1.5172	0.6306
	Yaw	3.2582	2.4932	1.0865

## Data Availability

The data presented in this study are available on request from the corresponding author. (The data are not publicly available due to privacy.)
